# Knockout of floral and meiosis genes using CRISPR/Cas9 produces male‐sterility in Eucalyptus without impacts on vegetative growth

**DOI:** 10.1002/pld3.507

**Published:** 2023-07-14

**Authors:** Michael F. Nagle, Surbhi S. Nahata, Bahiya Zahl, Alexa Niño de Rivera, Xavier V. Tacker, Estefania Elorriaga, Cathleen Ma, Greg S. Goralogia, Amy L. Klocko, Michael Gordon, Sonali Joshi, Steven H. Strauss

**Affiliations:** ^1^ Department of Forest Ecosystems and Society Oregon State University Corvallis Oregon USA

**Keywords:** biocontainment, CRISPR, Eucalyptus, flowering, forestry, male‐sterility, meiosis, pollen

## Abstract

*Eucalyptus* spp. are widely cultivated for the production of pulp, energy, essential oils, and as ornamentals. However, their dispersal from plantings, especially when grown as an exotic, can cause ecological disruptions. To provide new tools for prevention of sexual dispersal by pollen as well as to induce male‐sterility for hybrid breeding, we studied the clustered regularly interspaced short palindromic repeats (CRISPR)/Cas9‐mediated knockout of three floral genes in both *FT*‐expressing (early‐flowering) and non‐*FT* genotypes. We report male‐sterile phenotypes resulting from knockout of the homologs of all three genes, including one involved in meiosis and two regulating early stages of pollen development. The targeted genes were Eucalyptus homologs of *REC8* (*EREC8*), *TAPETAL DEVELOPMENT AND FUNCTION 1* (*ETDF1*), and *HECATE3* (*EHEC3‐like*). The *erec8* knockouts yielded abnormal pollen grains and a predominance of inviable pollen, whereas the *etdf1* and *ehec3‐like* knockouts produced virtually no pollen. In addition to male‐sterility, both *erec8* and *ehec3‐like* knockouts may provide complete sterility because the failure of *erec8* to undergo meiosis is expected to be independent of sex, and *ehec3‐like* knockouts produce flowers with shortened styles and no visible stigmas. When comparing knockouts to controls in wild‐type (non‐early‐flowering) backgrounds, we did not find visible morphological or statistical differences in vegetative traits, including average single‐leaf mass, stem volume, density of oil glands, or chlorophyll in leaves. Loss‐of‐function mutations in any of these three genes show promise as a means of inducing male‐ or complete sterility without impacting vegetative development.

## 1 INTRODUCTION

Eucalyptus is known for its fast growth and for providing diverse pulp, wood, energy, and oil products (Penín et al., 2020). It is also known for its ability to thrive in diverse and stressful climates far from its origins in and near Australia. Accordingly, Eucalyptus commonly spreads beyond exotic forest plantations and into wild ecosystems, where, in addition to the diverse economic and environmental benefits it can provide, it may also outcompete native trees, reduce water availability, impair local biodiversity, and otherwise disrupt regional ecosystems (Liang et al., 2016). Concerns about the dispersal of Eucalyptus, both when used as an exotic and in its home range, have been amplified in response to efforts to genetically engineer trees for desirable traits such as enhanced pest resistance (Ouyang & Li, 2016; Robischon, 2016) and frost tolerance (Hinchee, Zhang, et al., 2011). Thus, tools to mitigate its dispersal—such as via male‐ and/or complete sterility—may be critical to the continued or expanded use of Eucalyptus. An ablation‐based male‐sterility gene was developed during work towards commercializing cold‐tolerant Eucalyptus and wood‐dense pine in the United States (Hinchee, Rottmann, et al., 2011; Zhang et al., 2012), though possible vegetative effects have not been statistically evaluated to our knowledge.

Genetic sterility in trees can be induced using a number of tools from conventional breeding and biotechnology (Vining et al., 2012). The potential for sterility through the suppression or mutation of genes that are critical for reproduction has been made highly efficient by the development of gene editing using clustered regularly interspaced short palindromic repeats (CRISPR)/Cas9 systems (Goralogia et al., 2021). Previous work in Eucalyptus has demonstrated the viability of CRISPR/Cas9 for robustly producing knockout mutations and floral sterility. Knockout of *LEAFY* was accomplished with biallelic mutation rates near 100%, preventing the development of flowers while avoiding detectable pleiotropic effects on vegetative development (Elorriaga et al., 2021). We endeavored to build on this success by using CRISPR/Cas9 to target genes involved in later stages of sexual reproduction, with a focus on male‐sterility.

To select candidates likely to prevent the development of fertile flowers and pollen while avoiding pleiotropic effects on vegetative development, we surveyed published transcriptomes of *Eucalyptus grandis* floral and vegetative tissues (Vining et al., 2015). The first two candidates selected were putative functional homologs of the Arabidopsis genes *TAPETAL DEVELOPMENT AND FUNCTION 1* (*TDF1*) and *RECOMBINATION‐DEFICIENT 8* (*REC8*). The third candidate was a putative homolog of *HECATE 3* (*HEC3*).

*TDF1* encodes an R2R3‐Myb transcription factor that has been functionally characterized in Arabidopsis and is critical for normal development of the tapetum, a specialized layer of anther cells essential for providing nutrients, proteins, and nucleic acids needed for pollen development (Yao et al., 2022). In *tdf1* loss‐of‐function lines of Arabidopsis, over 1000 genes expressed in tapetal cells are significantly downregulated (Li et al., 2017). Among these differentially expressed genes are other transcription factors as well as E3 ligases and reactive oxygen species signaling proteins, noted for their roles in programmed cell death and callose dissolution, which are critical processes for the development of pollen grains (Li et al., 2017). In both Arabidopsis and rice, knockout of *TDF1* leads to phenotypes including excessive vacuolization and hypertrophy of tapetal cells, followed by the failure of pollen grains to develop, resulting in male‐sterility (Cai et al., 2015; Zhu et al., 2008). This considered, we hypothesized that knockout of the Eucalyptus *TDF1* homolog (*ETDF1*) would produce male‐sterility (via the absence of pollen) without vegetative defects. Our results largely supported this hypothesis, although one knockout line appeared to produce a very small amount of pollen.

*REC8* encodes a component of the cohesion complex essential for the cohesion of sister chromatids during meiosis. The function of *REC8* appears to be largely conserved across eukaryotes. Early studies of *REC8* were conducted using *Schizosaccharomyces pombe* (DeVeaux & Smith, 1994; Parisi et al., 1999) and *Saccharomyces cerevisiae* (Klein et al., 1999). *REC8* was later studied in *Mus musculus* (mouse), humans (Parisi et al., 1999), *Arabidopsis thaliana* (Bai et al., 1999; Cai et al., 2003), *Oryza sativa* (rice; Zhang et al., 2006; Mieulet et al., 2016; Shao et al., 2011), and *Citrullus lanatus* (watermelon; Cao et al., 2022). Across Arabidopsis, rice, and watermelon, loss of normal *REC8* function leads to disruptions of meiosis during pollen mother cell development, including disruption of pairing between homologous chromosomes and cohesion between sister chromatids, ultimately leading to unequal segregation of chromosomes, asymmetric tetrads, and inviable pollen (Bai et al., 1999; Cai et al., 2003; Cao et al., 2022; Mieulet et al., 2016; Shao et al., 2011; Zhang et al., 2006). In rice, suppression of *REC8* expression by RNA interference (RNAi) led to an approximate tenfold reduction in seed set in many RNAi lines, providing incomplete sterility (Zhang et al., 2006), whereas knockout of *REC8* was reported to produce completely infertile plants (Mieulet et al., 2016; Shao et al., 2011). Similarly, knockout of *REC8* in Arabidopsis was found to confer complete male‐ and female‐sterility (Bai et al., 1999; Cai et al., 2003). CRISPR/Cas9‐mediated knockout of *REC8* in watermelon resulted in seedless fruits, providing an alternative to the use of triploid lines (Cao et al., 2022). We expected knockout lines of the Eucalyptus *REC8* homolog (*EREC8*) to produce similar male‐ and female‐sterility. Viability staining and germination assays indicated that pollen in knockouts was entirely or almost entirely inviable. We were unable to assess female‐sterility in our experimental setting.

*HEC3* is part of a gene family with overlapping roles in regulating auxin and cytokinin signaling during reproductive development in Arabidopsis. The *HECATE* (*HEC*) family modulates the development of gynoecium tissues, including stigma and styles. Complete female infertility results from triple loss‐of‐function of *HEC1*, *HEC2*, and *HEC3* (in, while knockout or knockdown of certain individual or pairs of *HEC* genes leads to partial infertility. Among broad defects in the development of the inflorescence, knockouts of *HEC3* display abnormal phenotypes including reduced pollination and fertility (Gremski et al., 2007; Schuster et al., 2015). Although we hypothesized that knockout of the putative Eucalyptus homolog (*EHEC3‐like*) would produce similar morphological changes in female floral tissues, we report markedly distinct phenotypes, including the complete absence of visible stigma as well as male‐sterility.

Here, we report the efficient CRISPR/Cas9‐mediated knockout of the eucalypt homologs (*ETDF1*, *EREC8*, and *EHEC3‐like*) of each of these three gene targets. Because of low self‐fertility and poor adaptation to greenhouse conditions (where seed capsules were generally aborted before maturity), we were unable to derive reliable estimates of female‐fertility. However, male‐fertility is generally considered of greater importance than female‐fertility for genetic containment due to the potential for wide dispersal of Eucalyptus pollen by pollinators (Bezemer et al., 2016, 2019; Jones et al., 2008; Ottewell et al., 2009). Male‐sterility is also widely used to facilitate the production of hybrid crops (Wu et al., 2016) and thus may be useful for producing commercial eucalypt hybrids, which are widely grown (Potts & Dungey, 2004). We report that knockouts of each of the studied genes provided robust male‐sterility. Furthermore, considering the known functions and effects of homologs in other species, knockouts of two of these genes (*EREC8* and *EHEC3‐like*) may also provide strong female‐sterility. We did not find evidence for visible or statistically significant effects on vegetative development.

## 2 METHODS

### 2.1 Plant materials

**FIGURE 1:**
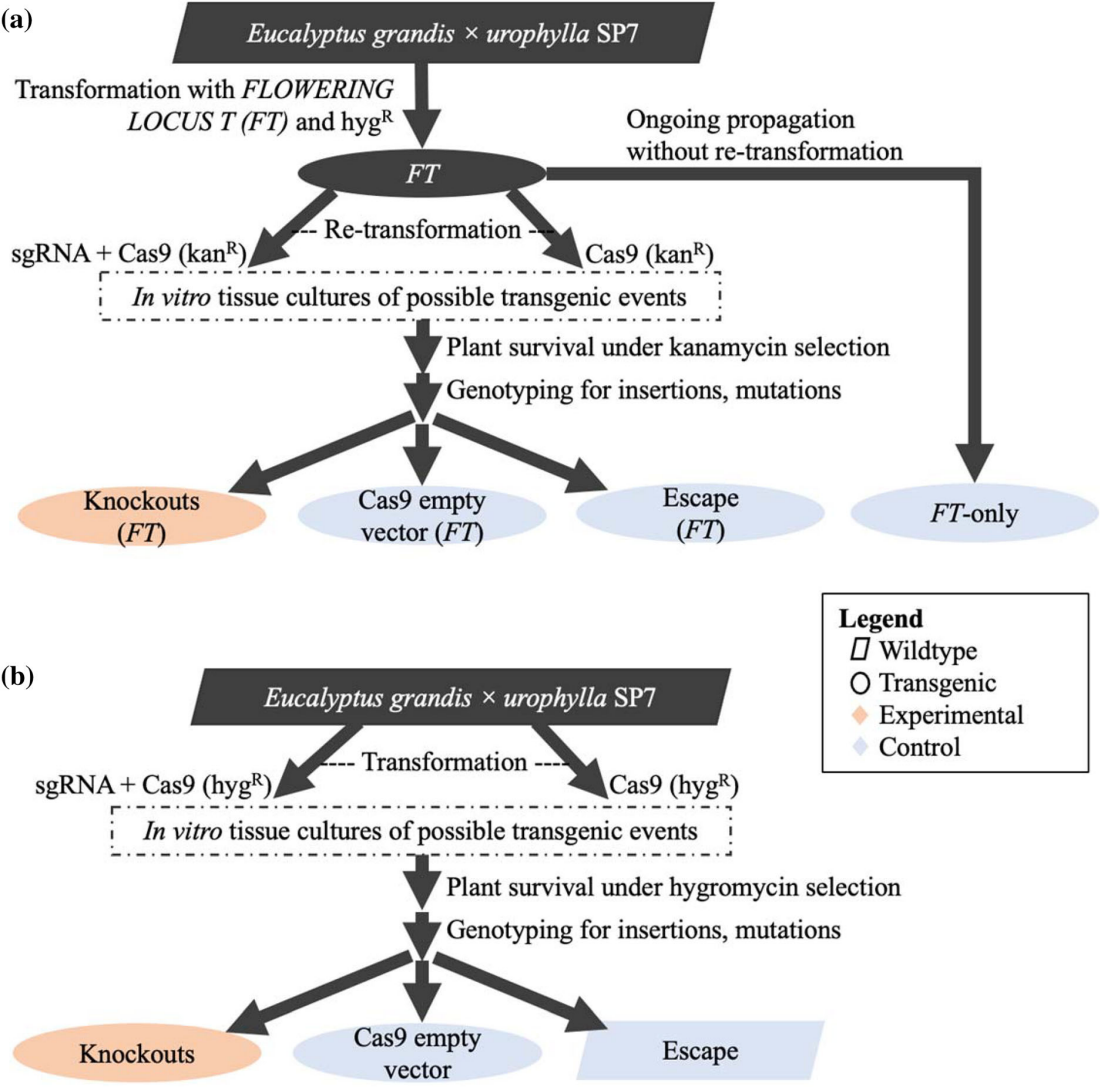
Schematic of experimental design and controls. We studied vegetative and floral effects of candidate gene knockout in two different genetic backgrounds of the *Eucalyptus grandis × urophylla* clone SP7. (a) Flowering traits were studied by retransformation of SP7 lines previously transformed to constitutively express Arabidopsis *FT* (Klocko et al., 2016). (b) Vegetative development was studied in a “wild‐type” SP7 background without constitutive expression of *FT*. The experimental plan is similar to that used by Elorriaga et al., 2021.

We studied the effects of gene knockout in the commercial hybrid Eucalyptus clone SP7 (*E. grandis × Eucalyptus urophylla*), generously provided by Futuragene/Suzano (Rehovot, Israel; Acknowledgments). This clone was micropropagated in sterile laboratory conditions, and the resulting plantlets were grown in a greenhouse at Oregon State University (East Greenhouse; approx. 44.567, −123.282). Our experimental design utilized the transformation of CRISPR/Cas9 constructs into two genetic backgrounds of SP7, with and without an early‐flowering trait. To support an accelerated study of fertility, we utilized early‐flowering lines featuring ectopic expression of Arabidopsis *FLOWERING LOCUS T* (*FT*). Because of the pleiotropic effects of the early‐flowering trait on vegetative development (Klocko et al., 2016), we also utilized a “wild‐type” SP7 background without this trait to study the effects of candidate gene knockout on normal vegetative growth. A schematic of the experimental lines and controls used in this work is provided in Figure 1.

### 2.2 Candidate gene selection

We first searched for candidate genes by cross‐referencing transcriptome data for floral and vegetative tissues in *E. grandis* (Vining et al., 2015), identifying genes that appear to be expressed exclusively in floral tissues in the transcriptome study. Arabidopsis homologs were then identified by Smith–Waterman peptide alignment data provided by Phytozome (https://phytozome-next.jgi.doe.gov). We also searched a transcriptome of male meiocyte expression in Arabidopsis (Libeau et al., 2011) for genes annotated as being involved in meiosis. For both groups, we performed a literature review on relevant genes in Arabidopsis and other species to identify candidates likely to produce sterile flowers without pleiotropic effects on vegetative development or the early stages of floral development. In an effort to avoid selecting target genes for which the loss of function might be compensated for by functionally redundant homologs, we searched for peptides predicted from the *E. grandis* reference genome (Myburg et al., 2014) via Basic Local Alignment Search Tool for proteins (BLASTp) for close homologs and selected only targets with distinctive conserved functional domains and protein structures. We next referred to *E. grandis* transcriptomic data for the selected genes to avoid targeting pseudogenes, ensure the genes have expected floral‐dominant expression patterns, and that any closely‐related paralogs are divergent in expression patterns. From these data, we selected our top three candidate genes to move forward with single guide RNA (sgRNA) design.

### 2.3 Selection of sgRNA sequences

CRISPRDirect (Naito et al., 2015) was used to search for protospacer adjacent motif (PAM) sequences and generate a list of possible sgRNA target sequences for each gene of interest, while avoiding sequences with four or more consecutive thymine bases (TTTT; which may cause premature termination) and seeking a GC content between 30% and 80%. Furthermore, this tool was used to filter out candidate sgRNA sequences that share more than 12bp of homology (adjacent to PAM) with any other sequences in the *E. grandis* genome. Next, sgRNAscorer (Chari et al., 2017) was used to rank candidate sgRNAs by predicted activity based on similarities to sgRNAs with experimentally confirmed activity (Table S1). Only exonic target sites were considered for sgRNAs. To enhance U6 promoter activity for each sgRNA, a 5′ G was appended to each if the sgRNA sequence did not already begin with a 5′ G (Ran et al., 2013). The selected target sequences are shown in Table S1.

Simple Modular Architecture Research Tool (SMART; Schultz et al., 1998) was used to determine the identities and positions of critical domains in the peptides encoded by each candidate gene and their putative homologs in *A. thaliana*. Using this information, we reduced our lists of candidate sgRNAs to prioritize those expected to produce mutations within or prior to essential functional domains and thus produce a complete loss‐of‐function mutation (Figure S1).

Finally, we sequenced portions of each gene in the *E. grandis × urophylla* hybrid clone SP7 used for transformation to ensure the sgRNAs used would effectively target both alleles in the genome (Figures S2–S4; primers in Table S2). Genomic DNA was extracted with a modified cetyltrimethylammonium bromide (CTAB) method adapted from prior work (Keb‐Llanes et al., 2002). Newly‐emerged leaves were collected from mature SP7 clones in the previously described greenhouse (see Section 2.1) in July 2016. These leaves were pulverized in liquid nitrogen using a mortar and pestle; insoluble materials were pelleted; and DNA was precipitated with ethanol, followed by washing. Isolated DNA was quantified with a NanoDrop spectrophotometer (Thermo Fisher Scientific), and approximately 50 ng was used for downstream PCR. We amplified regions of at least 200bp spanning each sgRNA via PCR with Q5 high‐fidelity polymerase (New England Biolabs) and submitted the purified amplicons for Sanger sequencing by Oregon State University's Center for Quantitative Life Sciences (formerly Center for Genome Research and Biocomputing). Primers used for PCR and Sanger sequencing are shown in Table S2. We evaluated the peaks in sequence data to ensure each fragment had at least one biallelic peak that demonstrated amplification of a natural SNP, indicating that both alleles had been amplified. We then replicated PCR and sequencing to make certain these biallelic peaks were not polymerase errors. Candidate sgRNAs with a biallelic peak within their sequence or the adjacent PAM site were excluded from consideration to avoid allele‐specificity.

### 2.4 Cloning of transformation constructs

Two sgRNAs were selected and cloned into given transformation constructs for each of our three target genes using a previously described cloning approach (Jacobs & Martin, 2016). We obtained oligonucleotides of 60nt in length or 61nt in cases where a 5′ G was appended to sgRNAs (Ran et al., 2013). For each sgRNA, oligonucleotides had 20bp of overlap with the U6 promoter, followed by the 20‐21bp sgRNA sequence (plus 5′ G when applicable), and followed by 20bp of overlap with the 5′ end of the sgRNA scaffold. These single‐stranded oligonucleotides were annealed and phosphorylated by co‐incubating forward and reverse oligonucleotide pairs (each at 100 μM) with T4 polynucleotide kinase (T4 PNK, New England BioLabs) on a thermocycler at 37°C for 30 min, followed by 95°C for 5 min, and finally a ramp down to 25°C by a 5°C decrease per minute.

The plasmids pUC Shuttle, p201N Cas9, and p201H Cas9 were ordered through Addgene (Jacobs et al., 2015). Constructs with sgRNAs were cloned by a Gibson Assembly/NEBuilder protocol as described previously (Jacobs & Martin, 2016). Using this method, we inserted two sgRNA cassettes for each target into SwaI‐ and SpeI‐digested p201N Cas9 (harboring kanamycin resistance for plants) and p201H Cas9 (harboring hygromycin resistance for plants). Successful insertions were confirmed by PCR and Sanger sequencing. An example construct map is presented in Figure S5.

Competent cells of *Escherichia coli* strain DH5α were transformed with each assembly product. To bulk plasmids and prepare for transformation of competent *Agrobacterium*, transgenic colonies were identified by PCR using primers ScaffoldF and MtU6R (Jacobs & Martin, 2016). The bulked plasmids were then transformed into competent Agrobacterium (strain AGL1) followed by plating on kan+ LB solid media. Plates were incubated for 2 days at 28°C and screened for colonies by PCR with the aforementioned primers. Molecular cloning was performed in 2016–2017, followed by plant transformation (2017–2018), micropropagation and screening of mutations (2017–2019), growth of plants in greenhouses, and phenotyping of greenhouse‐grown plants and their floral samples (2019–2021), as described below.

### 2.5 Plant transformation

Transformation constructs for *ETDF1*, *EREC8*, and *EHEC3‐like* sgRNAs and Cas9 were cloned as described above to produce versions of each harboring hygromycin and kanamycin selectable markers. The hygromycin‐selectable versions were used to transform a “wild‐type” commercial hybrid Eucalyptus clone SP7 (*E. grandis × urophylla*) generously provided by Futuragene/Suzano (Rehovot, Israel; Acknowledgments) as described previously (Klocko et al., 2016). This clone was micropropagated in sterile laboratory conditions, and the resulting plantlets were grown in the previously described greenhouse at Oregon State University. The plasmid p201H Cas9 was used to provide a hygromycin‐selectable “empty‐vector control” with no sgRNAs. Using versions of the transformation constructs with kanamycin selectable markers, we retransformed two lines of SP7 that were previously transformed with Arabidopsis *FLOWERING LOCUS T* (*FT*, already harboring hygromycin resistance) and thus flowered precociously (Klocko et al., 2016). The kanamycin‐selectable plasmid p201N Cas9 was used as an empty vector control for the *FT* background.

### 2.6 Screening for CRISPR/Cas9‐mediated mutations

Putatively transgenic plants surviving on kan+ (50 mg/L kanamycin) or hyg+ (5 mg/L hygromycin) shoot induction media were screened first by PCR to verify transgene insertion, followed by PCR to determine if each allele of the respective target gene was successfully mutated. PCR of transgene insertions was performed using two sets of primers for the Cas9 (product size: 324bp) and sgRNA cassettes (for transgenic events with experimental, non‐control plasmids; product sizes: ~170 and ~650bp). For Cas9 “empty vector” control constructs, primers used were p201R (near the Cas9 expression cassette) and StUbi3P218R (at the plant selectable marker expression cassette); and for constructs harboring sgRNAs, primers used were p201R (near the Cas9 expression cassette) and ScaffoldF (at sgRNA scaffolds; Jacobs & Martin, 2016).

Primers for PCR of each target gene are listed in Table S2 by sequence and in Table S3 by the event they were used for. Allele‐specific PCR was first attempted for all targets, and those presenting difficulties were sequenced with “universal” primers lacking allele‐specificity. In the case of one sample (*etdf1* event 5‐1 in the *FT* background), the amplicon was cloned into a sequencing vector prior to sequencing. Following PCR and gel purification of amplicons, sequences were determined by Sanger sequencing. The resulting sequences were aligned against wild‐type alleles using MEGA6 (Tamura et al., 2013). Using these alignments, specific insertions or deletions in CRISPR/Cas9 lines were determined relative to wildtype (Table S3).

### 2.7 Propagation and root induction

Following the identification of mutations by sequencing, shoots were propagated into deep Petri dishes (100 mm × 25 mm) with rooting media. After approximately 1 month, the plants were transferred from Petri dishes into soil pots in a headhouse, where they developed for three more weeks. Once transferred to pots, a Ziploc plastic bag was placed over the plants to retain moisture. The Ziploc bags were gradually opened in two increments (opened halfway for 2–3 days, and then opened completely) to allow gradual acclimation to ambient conditions in the headhouse. A version of this acclimation protocol was previously described for poplar (Meilan & Caiping, 2006). Throughout the processes of tissue culture and rooting, explants and ramets obtained via regeneration were kept indoors in a lighted growth room/headhouse at room temperature.

### 2.8 Acclimation in the greenhouse

**FIGURE 2:**
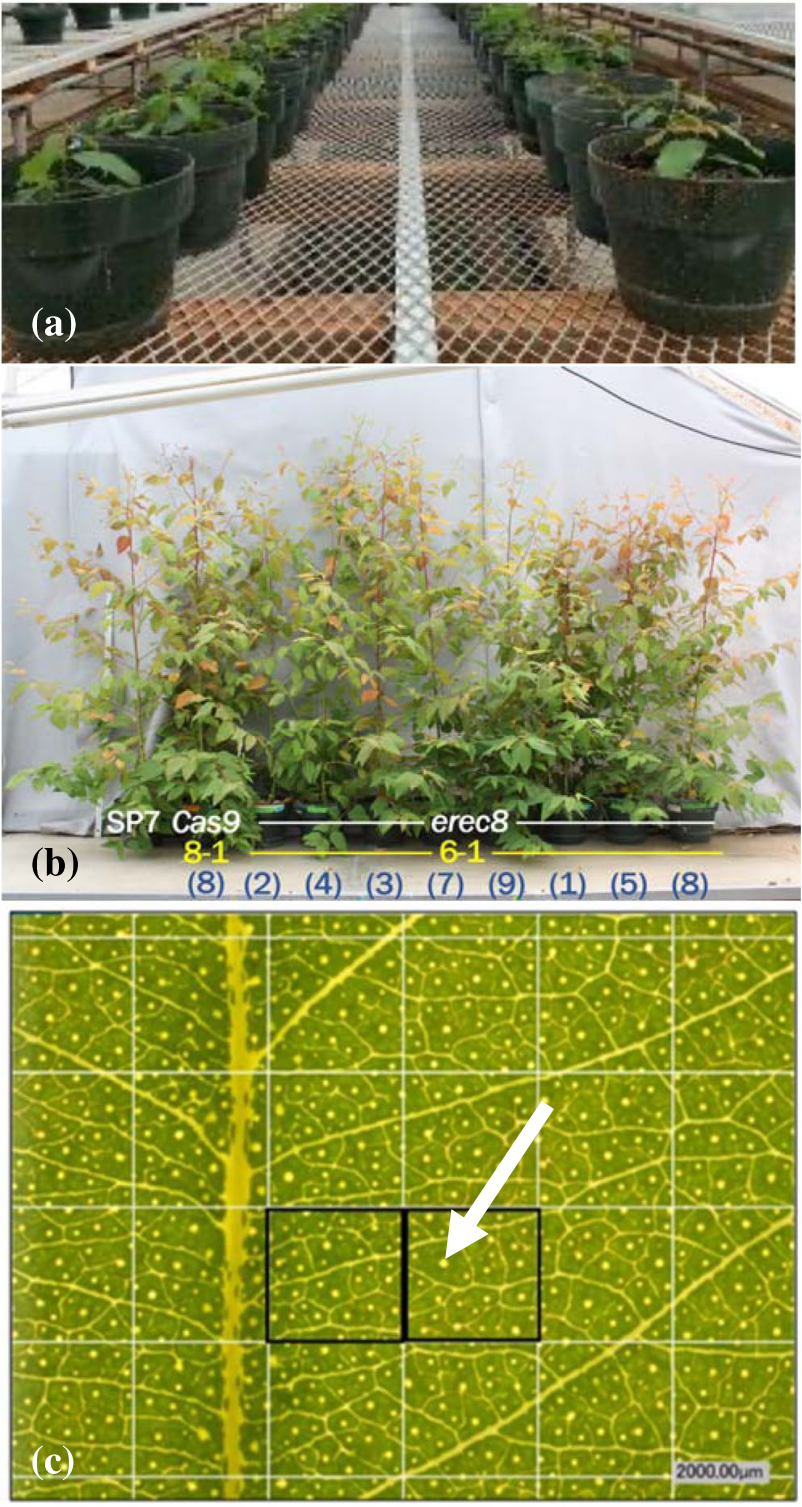
Selected components of phenotyping workflow. (a) View of plants shortly after being transplanted into large plots and transferred to the greenhouse. (b) View of plants after approximately 2 months of growth in the greenhouse. Randomized plants from a single knockout event were brought together for the photo, with SP7 and Cas9‐only controls on the far left. Genotype (white) is shown along with event number (yellow) and ramet identifier (blue). (c) An example of oil glands seen in the Keyence microscope at 30× magnification. Oil glands appear as approximately circular objects and a clear example is highlighted by white arrowhead. Two frames (e.g., as highlighted with black boxes) were used to estimate the density of oil glands in a leaf.

Following approximately 1 month of juvenile development in the headhouse, plants were transferred to the previously described greenhouse (Figure 2). When moving plants to the greenhouse, we transferred the plants into larger pots with slow‐release fertilizer in the soil. Plants that were first transferred and rooted tended to be larger when measurements began than plants that rooted later; thus, larger versus smaller plants were allocated to separate blocks. The greenhouse was kept at approximately 24–27°C with 16–8 h light–dark cycles. The numbers of events and ramets for sequence‐confirmed knockouts and control plants surviving greenhouse acclimation are listed in Table S4.

### 2.9 Vegetative phenotypes

Vegetative development was studied in SP7 plants without *FT* overexpression to avoid the strong influence of *FT* on plant size and branching (Klocko et al., 2016). We measured traits related to stem volume, leaf chlorophyll content, size, mass, and oil gland densities.

#### 2.9.1 Height and diameter

Plant height and diameter were measured twice, first at 2 weeks after plants were transferred into the large pots, and then at 3 months after the first measurement and towards the end of the experiment. Height was measured using a meter stick, and diameter was measured at approximately 5 cm above soil using Meba IP54 Electronic Digital Calipers. Plants were photographed at both points in time. The volume index was computed as height multiplied by the square of diameter.

#### 2.9.2 Leaf chlorophyll content

Relative chlorophyll content was determined using a SPAD 502 Plus Chlorophyll Meter (Konica Minolta Optics Inc.) based on three measurements per leaf on leaves collected from multiple locations on each plant. For the batch of plants including *etdf1* and *ehec3‐like*, samples were taken from three locations on each plant (near the base, middle, and top of the crown). For the batch with *erec8*, samples were collected from 40 cm above table height and from three separate sides of the plant. The mean of the readings was computed for each ramet (across nine observations total, three per each of the three leaves) and used for downstream statistical analysis. The leaf samples used for the measurement of chlorophyll content were also used for the measurement of leaf mass and area, as described below.

#### 2.9.3 Leaf area

A Scanjet 8200 scanner (HP Inc.) was used for leaf scans, where each scan included the three leaves from the same ramet and a ruler. Leaf area was calculated using ImageJ software (Schneider et al., 2012) with a scale based on the ruler. The image was converted to 8‐bit grayscale, and a threshold was tuned to distinguish leaves from the background. The “wand” tool was used to outline each of the three leaves; areas were then calculated and recorded. For each ramet, the mean leaf area was computed and used for downstream statistical analysis.

#### 2.9.4 Leaf mass (dry weight)

After measuring leaf area, leaves were placed in a drying oven at 62°C for 10–12 days. All three leaves from each given ramet were weighed together on a Mettler AJ100 scale. Mean weights for each ramet were computed.

#### 2.9.5 Leaf oil gland density

For measurement of oil gland density, new leaves were collected. For the batch of plants with *etdf1* and *ehec3*‐*like*knockout lines, leaves were collected at approximately 15 cm from the tip of a branch, near the base of the plant. For the batch with *erec8*, leaves were collected approximately 15 cm from the tip of a branch that was approximately 50 cm from the top of the pot. Oil gland density in these fresh leaves was evaluated using a Keyence VHX‐1000E digital microscope. Leaves were placed on the microscope platform with the adaxial surface of the leaf facing upward towards the lens. Two cells from a 4 × 4 grid were pseudo‐randomly selected from the middle of the leaf area (while excluding the midrib) and used to count the number of oil glands per unit area (Figure 2c). Means for each ramet were used for statistical analysis.

### 2.10 Flowering and reproductive trait phenotypes

#### 2.10.1 Pollen collection

Pollen production and other floral traits were studied in the *FT* background. To select floral buds for pollen collection and staining, we began by identifying mature buds that were close to flowering, as indicated by the outer operculum beginning to shed. Stems with soon‐to‐flower buds were collected and placed in 15 or 50 mL Falcon tubes with water, without submerging the buds in water. Two buds were collected and studied for each selected ramet. These tubes were stored on a rack, placed in a shallow water bath, and incubated overnight at 28°C. The following day, flowering buds were collected, and scissors were used to remove the anthers, which were placed into tin‐foil packets. These packets were left in a desiccator containing silica beads for approximately 48 h. Afterward, the contents of the packets were transferred to microcentrifuge tubes for immediate staining, or alternatively, packets were placed in glass jars with silica beads and stored at 4°C for staining or germination tests at a later time.

#### 2.10.2 Pollen staining

In all cases, staining was completed within 1 month of harvesting anthers, and both transgenics and controls were assayed at similar times. The staining solution was prepared according to a previously‐described pollen viability staining method (Peterson et al., 2010). Anthers were vortexed vigorously in microcentrifuge tubes for 30 s. Next, 400 μL of staining solution was added to each microcentrifuge tube, and the tubes were shaken by hand. Each sample was incubated in a hot water bath at 55°C for 4 min. To separate pollen from anther debris, the solution with sample contents was pipetted through a 30 μm mesh cloth into a fresh tube. The original tubes with the remaining anther debris were discarded. The cloths were left on top of the fresh tubes, and the lids were closed so that the cloth was clamped underneath. Samples were centrifuged at 1000 RPM using a full‐size tabletop centrifuge for approximately 1 min. Mesh clothes were removed, and samples were again centrifuged at 1400 RPM for approximately 2 min, resulting in a pollen pellet. In cases in which no pollen pellet was clearly visible at this stage, centrifuging was continued incrementally until a pellet appeared. After centrifugation, the supernatant was removed by pipette, taking care to avoid disturbing the pellet. Next, 24 μL of a 50:50 (by volume) water/glycerin solution was added to each sample, followed by shaking the tubes by hand to resuspend pellets. Samples with a distinctly purple color remaining were washed by re‐pelleting, removal of the supernatant, and replacement with a fresh water/glycerin solution.

From each sample, 10 μL was pipetted onto a hemocytometer slide and viewed under a Keyence VHX‐1000 digital microscope. Each image was divided according to a 4 × 4 grid, and four grid cells were randomly selected for counting viable and total pollen. The concentrations of viable and total pollen per μL were calculated. Then, using the analyzed sample volume and the total volume, we estimated the count of viable and total pollen per bud.

#### 2.10.3 Pollen germination assay

For pollen‐producing *erec8* in the *FT* background, along with *FT* control plants, we next performed pollen germination assays using unstained aliquots of pollen isolates from our greenhouse. A protocol was provided courtesy of Nicky Jones from Sappi (personal communication; Acknowledgments) and was performed as follows: Upon removal from storage, anthers were transferred into 15 mL Falcon tubes and vortexed on high for 30 s to dislodge pollen. Vortexed samples were then flooded with 400 μL of pre‐germination liquid media (15% sucrose, .01% boric acid, .03% calcium nitrate tetrahydrate, .02% magnesium sulfate heptahydrate, and .01% potassium nitrate) and gently hand‐shaken to promote mixing of pollen. The remaining solution was pipetted from the 15 mL Falcon tube into a 2 mL microcentrifuge tube through a 30 μM filter cloth. Microcentrifuge tubes with filtered pollen mixtures were centrifuged for 1 min at 1000 RPM. The filter was removed, and samples were centrifuged again at 1400 RPM for 2 min to form a pollen pellet. The supernatant was removed by pipetting, leaving only the pollen pellets. Pellets were then flooded with 24 μL of new pre‐germination liquid media and mixed by pipetting. Ten microliter aliquots were pipetted onto Petri dishes containing germination solid media (containing 1% agar in addition to the same components as previously described liquid media). The Petri dishes were then incubated at 20°C and imaged first at 18 h and twice again at 6 h intervals. Pollen germination was scored using a visual scale with four discrete values from 0 to 3, representing the extent of germination observed (Figure S6).

### 2.11 Statistical analysis

Because our evaluation of vegetative development was conducted at two different times of year and by different personnel using somewhat different sampling criteria (one for *etdf1* and *ehec3‐like* and their controls, and another for *erec8* and its controls), we performed statistical analysis separately for these two batches. Included in our analyses were two types of controls for each batch: (1) plants that survived selection on hyg+ media despite possessing no transgene insertion as determined by PCR (termed “escapes”); and (2) plants confirmed to contain the control plasmid, p201H Cas9, possessing the Cas9 and hygromycin resistance genes with no sgRNA cassettes (Figure 1). First, we visualized the effects of constructs on each trait while adjusting for block effects; the block adjustment was performed by subtracting mean effects for each trait in each block from trait values, followed by re‐scaling by adding the overall mean for each trait. We also visualized relationships between traits with correlation plots. Plots revealed an outlier event with an unusual mutant phenotype that was removed prior to continuing analysis (see Data S1). Next, we evaluated the viability of a mixed linear model (MLM) for modeling traits of interest without any transformation of traits. Towards this end, we constructed a MLM for each batch, in which the fixed effects were block and transformation construct (or lack thereof, in the case of “escape” controls), and the random effect was the transformation event, nested within transformation construct. Residual plots were examined to determine whether transformations of traits were necessary to avoid violations of MLM assumptions. Two traits displayed heteroscedasticity in residual plots (volume index and leaf mass) and were therefore log‐transformed. Subsequently, MLM construction and graphical evaluation were repeated for these two traits to ensure that the assumption of homoscedasticity was not violated (Figures S7–S10).

MLMs using these data with transformations for particular traits were used for downstream analyses with F‐tests and estimated marginal means (EMM) using base R and the “emmeans” package, respectively. EMM analysis was performed to find differences between pairs of constructs (or between a given construct and the “escape” control). Confidence intervals (CIs) were computed for EMM results to determine whether 95% CIs included zero, which would suggest a lack of difference between pairs. We also computed the mean difference between pairs, regardless of whether the difference was significant or not. The values of CIs were converted from a scale of the trait value to a percentage change relative to each type of control (p201H Cas9 control and “escape” control). For both F‐test and EMM results, *p*‐values were also computed for effects of genotype class (including gene knockout treatments and each type of control).

Pollen number and viability results comparing *etdf1* and *ehec3‐like* to controls were qualitative (zero in knockouts, with the exception of one *ehec3‐like* event with an extremely small number of pollen grains). A simple Student's *t*‐test was applied to the style length of *ehec3‐like* and control flowers. Germination rates for *erec8* and control pollen were studied with a Fisher's exact test. The Student's *t*‐test and Fisher's exact test were performed using Microsoft Excel functions.

## 3 RESULTS AND DISCUSSION

### 3.1 Sequence alignment and gene expression informs homology of gene targets

**FIGURE 3:**
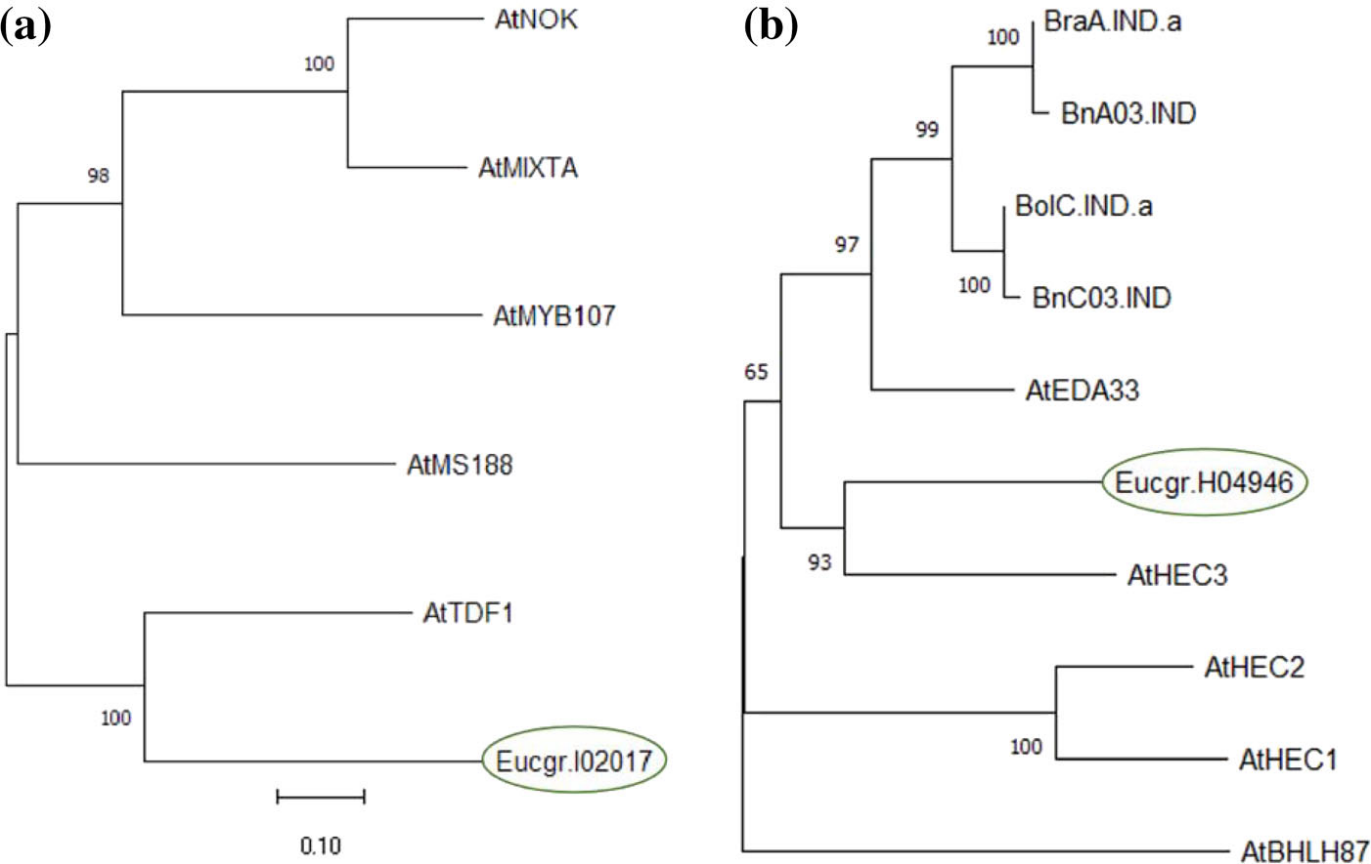
Phylogenetic relationships of target genes to close homologs. (a) ETDF1 (Eucgr.I02017), (b) EHEC3‐like (Eucgr.H04946) and possible homologs of each, were analyzed by MUSCLE alignment of predicted peptide sequences and nearest‐neighbor joining by bootstrapping. Numerical values on branchpoints represent bootstrap scores with 1000 bootstrap replications. Branch lengths represent nucleotide substitutions per site, with a scale bar for reference. The Eucalyptus homologs studied in this work [ETDF1; (Eucgr.I02017) and EHEC3‐like (Eucgr.H04946)] are circled in green.

Alignment of the ETDF1 predicted peptide against peptides predicted from the *Arabidopsis thaliana* genome, both via Smith–Waterman (Table S5A) and BLASTp (Table S6A), indicated that this gene target is a member of a Myb‐domain protein family expressed during floral development, with critical roles in the development of the tapetum and pollen. Arabidopsis homolog annotations were obtained from The Arabidopsis Information Resource (arabidopsis.org). The most closely related ortholog in *A. thaliana* is AtTDF1 (score = 234; similarity = 83.6%). Other closely related *A. thaliana* orthologs include the tapetal development protein MALE STERILE 188 (AT5G56110; score = 218, similarity = 78.2%; Li et al., 2007), the trichome and cuticle development protein AtMIXTA (AT5G15310; score = 198; similarity = 81.3%; Camoirano et al., 2020), and a regulator of biosynthesis of the seed coat polymer suberin, MYB107 (AT3G02940; score = 195; similarity = 82.8%; Gou et al., 2017). Although members of this gene family are closely related, the two most closely related to ETDF1 in terms of Smith–Waterman alignment score are both essential for male‐fertility in Arabidopsis (Li et al., 2007; Zhu et al., 2008). Phylogenetic analysis, including predicted peptides for the five most closely related Arabidopsis genes, suggested that all except ETDF1 are members of a clade distinct from AtTDF1 and ETDF1 (Figure 3a).

Smith–Waterman alignment data available from Phytozome lists AtREC8 as the only *A. thaliana* homolog of EREC8 (score = 217, similarity 56.1%). We performed BLASTp alignments (Table S6B) to investigate whether there could be additional homologs that do meet the thresholds required for the Smith–Waterman alignment. BLASTp yielded two aligned segments between EREC8 and AtREC8, including a 142‐residue length alignment including a portion of the N‐terminal domain and a 349‐residue length alignment with a portion of the C‐terminal domain. No alignment with any other *A. thaliana* peptide was of a length greater than 64 residues. Alignments with proteins other than REC8 are with other cohesin complex proteins, notably distinct from REC8 due to their vegetative expression and likely distinct roles in sister chromatid cohesion in mitosis (Figures S11–S14; da Costa‐Nunes et al., 2006).

Results from Smith–Waterman alignment (Table S5B) and BLASTp (Table S6C) did not provide clear evidence of whether EHEC3‐like is more likely to share function with INDEHISCENT1/EMBYRO DEVELOPMENT ARREST 33 (IND1/EDA33; AT4G00120; score = 156; similarity = 86.7%) or the HEC protein family (with scores ranging from 126 to 149, similarity ranging from 89.2% to 91.3%). We also note that among the top five most closely related Arabidopsis predicted peptides is an uncharacterized bHLH protein (AT3G21330; score = 132; similarity = 75.5%). For further insight, we constructed a phylogram with these homologs as well as *Brassica* spp. homologs of IND1/EDA33. We found a distinct clade, including AtHEC3 and our candidate gene (Figure 3b).

Finally, we consulted Smith–Waterman and BLASTp alignments of each of these three candidate genes against the genome of *E. grandis*, and merged these results with gene expression data for *E. grandis*, obtained by RNA‐Seq in a prior study (Tables S7 and S8; Vining et al., 2015). No closely related peptides were found in the *E. grandis* reference for EREC8. Smith–Waterman alignments for ETDF1 and EHEC3‐like, as well as BLASTp alignments for all three predicted peptides, did not reveal any closely‐related paralogs with similar expression patterns that showed a preference for floral development, suggesting a lack of functional redundancy. These results suggested that knockout of each gene target would likely result in a loss‐of‐function phenotype rather than being compensated for by redundant paralogs.

The previously published RNA‐Seq data leveraged in this work features samples from two broad stages of floral development, informing that the expression of our candidate genes is mostly or entirely specific to floral bud tissues at early or late stages of development (Vining et al., 2015). Future research on each of these candidate genes will benefit from *in situ* hybridization to inform the detailed timing and cytological patterns of gene expression across specific floral tissues, as well as the extent to which these mirror or differ from expression in Arabidopsis and other species.

### 3.2 Efficient knockout of genes targeted with CRISPR/Cas9

We determined mutations in CRISPR/Cas9 lines relative to wildtype via sequence alignment and recorded the lengths and types (e.g., insertion, deletion, and inversion) of mutations in each event (Table S3). Summary statistics of mutation rates (Table S9) were computed for all samples for which both alleles were successfully sequenced, with results summarized as follows. Mutations of each sgRNA site with CRISPR/Cas9 were of moderately high efficiency overall, with mutations detected at 69.83% of possible mutation sites evaluated (overall mutation rates). The usage of two sgRNAs per gene increased the effective mutation rate per gene substantially, as 85.34% of alleles (across genes and samples) were mutated in at least one of the two sgRNA sites. Our objective in using CRISPR/Cas9 was to produce knockouts of each gene target via large deletions and/or out‐of‐frame mutations that lead to missense and nonsense of protein sequences prior to essential functional domains. However, because a portion of mutations yielded short and in‐frame modifications, only 70.69% (out of the total events sequenced) were expected with high confidence to produce knockout phenotypes.

Effective mutation rates (by allele and across sgRNAs) varied across gene targets, ranging from 75.0% for *etdf1* to 90.48% for *erec8* and 94.44% for *ehec3‐like*. These rates do not show clear agreement with predicted sgRNA activities, as *EREC8* sgRNAs were predicted to have the greatest activity while those of *ETDF1* and *EHEC3‐like* were roughly similar (Table S1). The most common mutations were simple deletions (defined as a deletion spanning one or both sgRNA sites without an additional insertion or inversion), which were observed at 49.14% of possible mutation sites. Simple insertions were observed at 14.22% of sites (33 sites). Among these, 31 had single‐base insertions, and two were of much greater length (498bp and 530bp), both of which were observed with the second sgRNA targeting *EHEC3‐like*.

We found mutations spanning both sgRNA sites of a given allele in a given sample at a low rate (24.14%). These included large deletions as well as inversions and compound mutations of a mixed nature spanning the area of several hundred bases between two sgRNA sites. We did not observe a relationship between the rate of these events and the distance between sgRNA target sites; this rate was 11.90% for *erec8* (~290bp between PAM sites), 19.44% for *etdf1* (~840bp between PAM sites), and 44.44% for *ehec3‐like* (~150bp between PAM sites). In some cases, these biallelic mutations spanning both sgRNA sites were observed as simple inversions of the nucleotide sequence between sites (4.31% of alleles across genes and samples). We also observed three cases of complex mutations involving combinations of insertions, deletions, and/or inversions, two of which were found to span two sites. When large mutations spanned both sgRNA sites, we assumed that these represented nuclease activity occurring at both sites, although we note a lack of certainty in this inference.

We compared our results to several published reports involving the use of multiple sgRNAs to target given genes, in particular comparing biallelic mutation rates, defined for our purposes as the rate at which both alleles of a gene are mutated at one or more sgRNA sites. The rates we observed were lower than those in a previous report of highly efficient CRISPR/Cas9‐mediated mutagenesis for the eucalypt homolog of the flowering gene *LEAFY* (Elorriaga et al., 2021). In that study, almost 100% of samples were found to have biallelic mutations. We report the same statistic to be 85.34% among our gene targets, using a CRISPR/Cas9 and dual‐sgRNA system that was similar but notably different in that sgRNA expression was driven by the *Medicago truncatula* U6 promoter (Jacobs et al., 2015), whereas this prior study utilized the U6 promoter from Arabidopsis (Elorriaga et al., 2018, 2021). Another study using CRISPR/Cas9 with two sgRNAs in Eucalyptus reported rates of 4.2% for one gene and 84.6% for another (Dai et al., 2020). In citrus, CRISPR/Cas9‐mediated editing produced a biallelic mutation rate of 55.5% with the use of three sgRNAs targeting a single gene (Huang et al., 2020). These results, as well as our own, highlight the wide variation in mutation efficiency across sgRNA sites and genes, which we expect is the result of a combination of sgRNA binding efficiency together with chromatin accessibility (Jensen et al., 2017; Liu et al., 2019; Weiss et al., 2022; Yarrington et al., 2018). Although there exist many approaches to test sgRNA activity via transient or in vitro assays (Fister et al., 2018; Liang et al., 2019; Liu et al., 2018; Shkryl et al., 2021), doing so is not essential; we find that the use of two sgRNAs per target appears to work well, acting as a hedge against the possibility of inefficient activity of any single given sgRNA. The dual‐sgRNA approach also provides the advantage of occasionally yielding large deletions that span two sgRNA sites and are thus easy to assay on gels during further analysis, and when spanning essential functional domains, they are very likely to produce strong and stable loss‐of‐function phenotypes.

### 3.3 Absence of clear pleiotropic effects of target gene knockout on vegetative development

**FIGURE 4:**
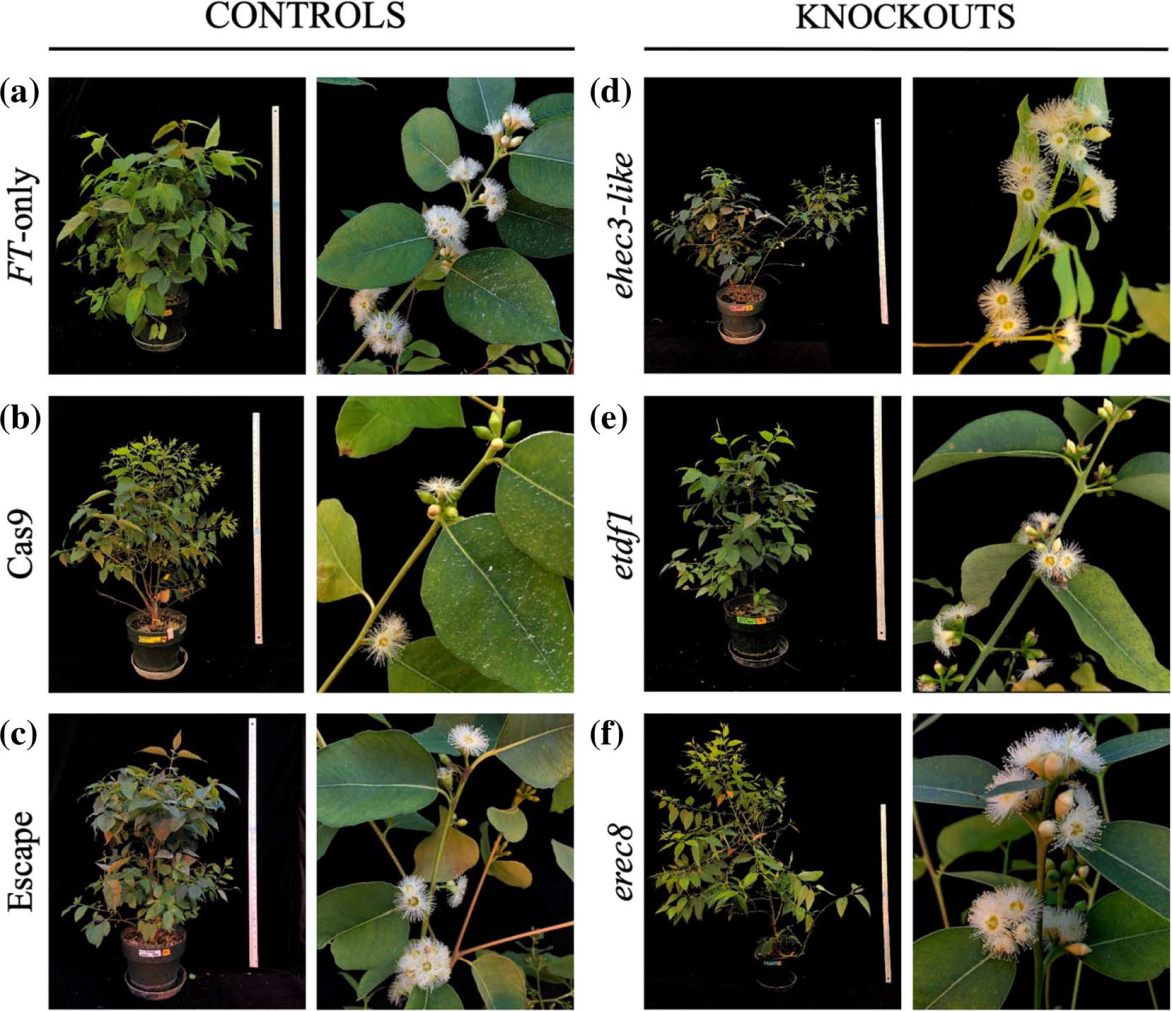
Representative whole trees (left) from each control and knockout, with examples of flowering sections from each tree (right). (a) *FT*‐only controls, line 4‐2 [left: ramet 107, right: ramet 3]. (b) Cas9 controls, event 15‐2 (left) and 1‐2 (right) [left: ramet 124, right: ramet 122]. (c) Escape controls, line 4‐2 [left: ramet 53, right: ramet 12]. (d) *ehec3‐like*, event 18‐2 (left) and 16‐1 (right) [left: ramet 82, right: ramet 72]. (e) *etdf1*, event 28‐1 (left) and 6‐1 (right) [left: ramet 19, right: ramet 38]. (f) e*rec8*, event 29‐1, ramet 120 (left and right).

An informal visual comparison of knockout and control plants did not reveal any obvious morphological differences (Figure 4). Statistical analysis was performed to inform whether variations existed for measured traits, including leaf mass and area, oil gland density (Figure 2c), leaf chlorophyll content (SPAD reading), plant height, stem diameter, and stem volume index. F‐tests and EMM analyses did not reveal any significant effects of target gene knockouts on vegetative traits, given the appropriate handling of confounding influences and outliers (Tables 1, S10, S11, and S12). As detailed in Data S1, the batch of plants including *etdf1*, *ehec3‐like* and their Cas9 and “escape” controls was subject to a confounding influence because these “escape” controls were rooted 2 weeks prior to the other plants, and initial size at the time of rooting correlated with vegetative traits of interest. No statistically significant (*p* < .05) effects were detected when this influence was avoided by excluding the “escape” controls. However, effects on leaf mass and oil gland density in the *etdf1*/*ehec3‐like* group were suggested (*p* < .10) both by F‐tests and EMM tests, indicating that further research into whether one or both of these genes can affect these traits when mutated would be prudent. No statistically significant or near‐significant effects of *erec8* knockout were detected for the batch with *erec8* plants and controls, regardless of analysis method.

**TABLE 1:** F‐test *p‐*values for selected ANOVA models. Candidate genes and respective controls were divided into two groups for practical evaluation. We performed several rounds of analysis while determining the appropriate handling of controls for each group (materials and methods). Our evaluations of vegetative traits involved up to two types of controls (Figure 1). Presented are selected models for the group with *erec8*, Cas9 control, and escape control plants (initial model for this batch; Table S10B) and the group with *etdf1*, *ehec3‐like*, and Cas9 control plants (second model, excluding the escape control due to it being propagated at a different time; Table S11A). Statistically significant effects (*p <* .05) are shown in italics.

Group	*p*‐values							
	Variable	Leaf area	Leaf mass	Diameter	Height	Volume index	Oil gland density	SPAD
*erec8*, Cas9 control, escape control	Block	*8.4E‐12*	*.0017*	*.0346*	*2.5E‐13*	*3.3E‐06*	.5572	*.0362*
	Genotype class	.6264	.3132	.9281	.2694	.8259	.9798	.3847
*etdf1*, *ehec3‐like*, Cas9 control	Block	.1473	.1641	.2170	*1.7E‐05*	*.0073*	.2079	*1.3E‐04*
	Genotype class	.4499	.0627	.1082	.6706	.1733	.0866	.5403

In conclusion, our tests did not provide evidence for a clear and statistically significant effect of knockout of *ETDF1*, *EHEC3‐like*, or *EREC8* on any measure of vegetative development in the greenhouse. However, further research involving larger sample sizes and field trials will be needed to determine conclusively whether knockout of these genes can provide sterility while avoiding all pleiotropic effects on biomass growth or other vegetative traits important to forestry.

### 3.4 *ETDF1* is necessary only for pollen development, similar to Arabidopsis *TDF1*

**FIGURE 5:**
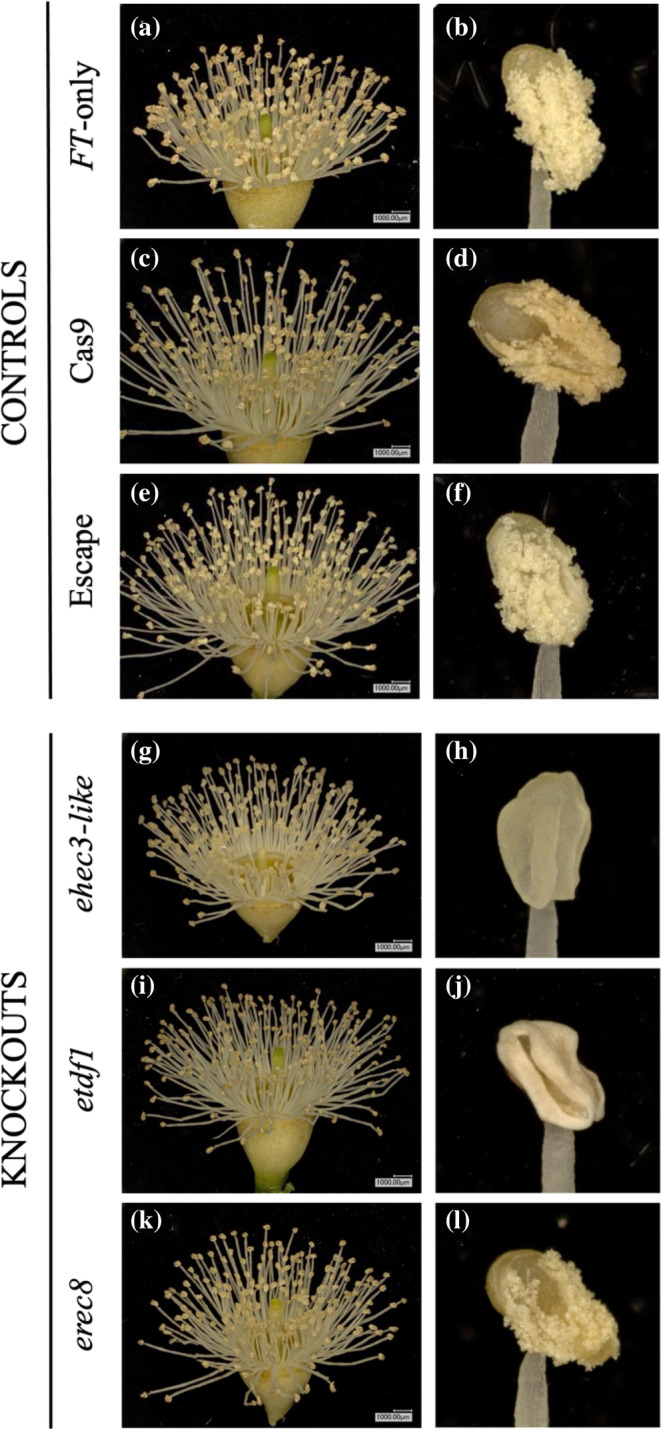
Absence of visible pollen on flowers and anthers in *ehec3‐like* and *etdf1* lines. (a, b) Flower and anther from *FT*‐only control, line 4‐2 [A, B: ramet 74]. (c, d) Flower and anther from Cas9 control, event 15‐2 [C: ramet 124, D: ramet 61]. (e, f) Flower and anther from escape control, line 4‐2 [E, F: ramet 106]. (g, h) Flower and anther from *ehec3‐like*, event 16‐1 [G, H: ramet 60]. (i, j) Flower and anther from *etdf1*, event 6‐1 [I: ramet 99, J: ramet 38]. (k, l) Flower and anther from e*rec8*, event 34‐2 [K, L: ramet 123].

**FIGURE 6:**
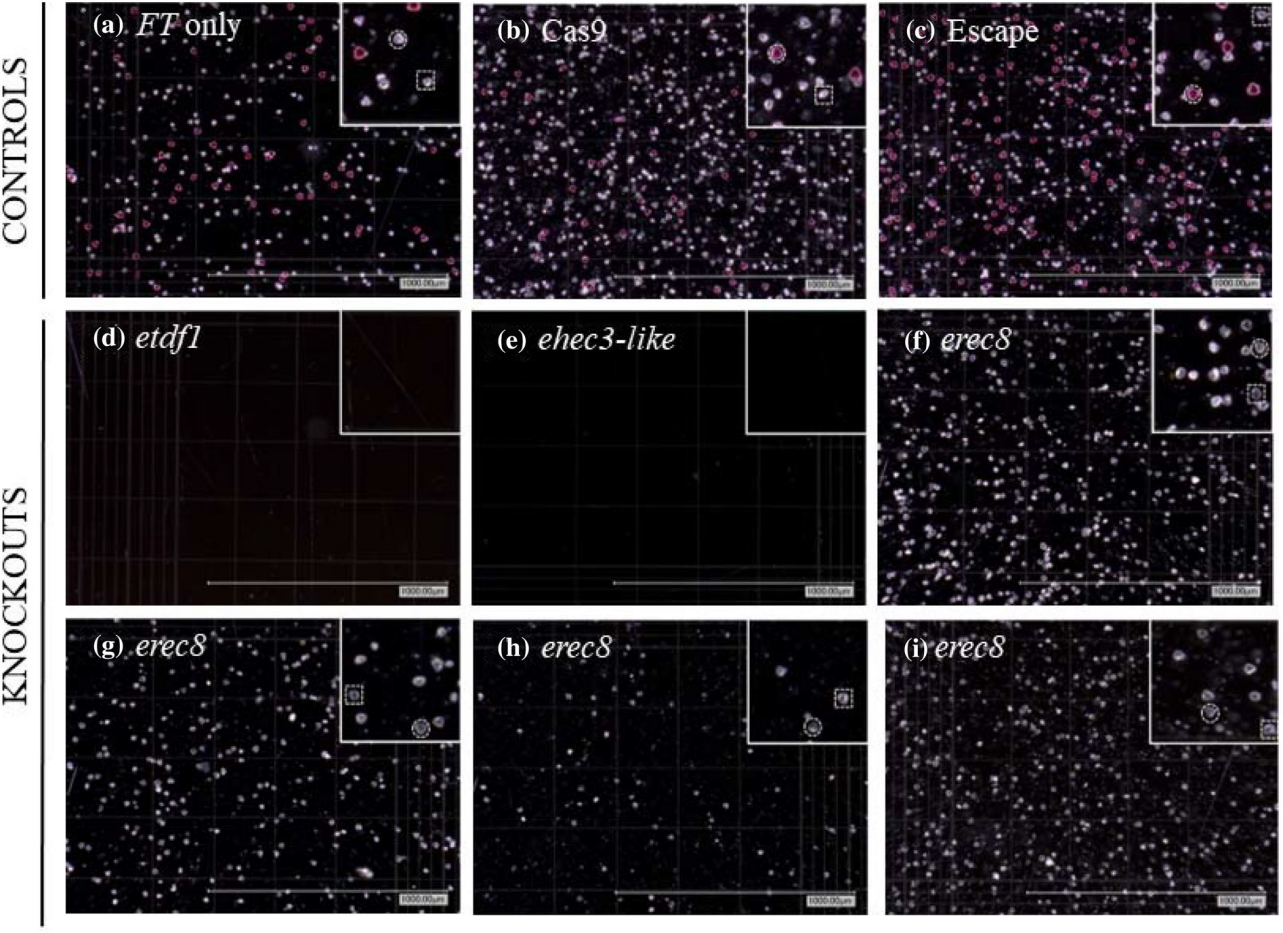
Pollen yield, morphology, and viability of knockout and control samples. Staining, microscopy, and counting of pollen allowed the estimation of viable pollen yields. Putatively viable pollen appears red with Alexander stain. Four events are shown for *erec8*, while a single event is shown for the pollen‐lacking *etdf1* and *ehec3‐like*. Controls and events selected have IDs as follows: (a) ramet 95, *FT*‐only line 4‐2, (b) ramet 124, Cas9 event 15‐2 in *FT* line 30‐3, (c) ramet 106, escape of *FT* line 4‐2, (d) ramet 38, *etdf1* event 6‐1 in *FT* line 30‐3, (e) ramet 60, *ehec3‐like* event 16‐1 in *FT* line 4‐2, (f) ramet 26, *erec8* event 8‐2 in *FT* line 30‐3, (g) ramet 133, *erec8* event 29‐1 in *FT* line 30‐3, (h) ramet 22, *erec8* event 18‐2 in *FT* line 4‐2, (i) ramet 127, *erec8* event 34‐2 in *FT* line 4‐2.

In the SP7 commercial hybrid of *E. grandis × urophylla*, we identified an *etdf1* phenotype involving loss of pollen without any other macroscopic changes in the development of either flowers or vegetative tissues. Pollen was not visible by eye on anthers (Figure 5), nor by microscopy following our pollen collection protocol (Figure 6, Table S13). The lack of pollen is consistent with reports in Arabidopsis and rice where loss of functional *TDF1* leads to male‐sterility due to a failure to produce pollen grains. In both Arabidopsis and rice, *TDF1* has been demonstrated to act upstream of a range of genes involved in tapetal development, and failure of pollen development in loss‐of‐function lines is believed to result from vacuolization and hypertrophy of tapetal cells (Cai et al., 2015; Zhu et al., 2008).

The pattern of *ETDF1* expression is generally aligned with that in Arabidopsis, although the Eucalyptus floral transcriptome is of limited resolution across tissues and we lack data for expression specific to pollen or tapetal cells. RNA‐Seq data for *E. grandis* indicates that *ETDF1* expression becomes detectable during early flower development (where floral buds become distinguishable from vegetative buds) and increases in late flower development (where floral bracts have shed). *ETDF1* transcripts were not detected during any vegetative stages of development (Table S14; Vining et al., 2015). The apparent specificity of *ETDF1* to male floral development without vegetative roles agrees well with current models for *TDF1* function in Arabidopsis and rice. In Arabidopsis, *TDF1* expression is reported to be found only in floral tissues. A study using RNA‐Seq data detected floral expression beginning around the time of bud opening (Klepikova et al., 2016), while a study using *in situ* hybridization revealed floral expression near the time of microspore mother cells entering meiosis. The aberrant phenotypes resulting from *TDF1* knockout appeared first as vacuolization of cells in the epidermis and the endothecium of developing anthers (Zhu et al., 2008). *TDF1* is a member of a transcription factor network regulating tapetum development (Li et al., 2017; Zhu et al., 2011) and is also enriched in male meiocytes (Libeau et al., 2011). In rice, a *TDF1* homolog is similarly expressed in anthers, and loss‐of‐function results in similar phenotypes of vacuolization and male‐sterility (Cai et al., 2015).

Because the scope of our work was limited to exploring the potential biotechnological utility of *ETDF1* and other candidate genes in producing sterile Eucalyptus, we did not perform cytological and mechanistic analyses to determine if the failure of pollen development is associated with vacuolization and hypertrophy in the tapetum as in other species (Cai et al., 2015; Zhu et al., 2008). However, the complete or near‐complete absence of pollen in the knockout lines we studied, together with the floral specificity and sequence similarity of *TDF1* and orthologs across species (Tables S5, S6, and S14), suggest that the primary mechanistic role of *TDF1* is likely conserved. Further research involving cytological analysis of anther sections may confirm this and provide mechanistic insights necessary for a better understanding of *ETDF1* function and well‐informed biotechnological applications of this gene target. Moreover, investigating Eucalyptus homologs of upstream regulators and downstream transcriptional targets of Arabidopsis and/or rice TDF1 (Cai et al., 2015; Li et al., 2017; Zhu et al., 2011) will inform about the extent to which the genetic regulatory network of tapetum development is conserved.

### 3.5 *EREC8* is necessary for meiosis and expressed in vegetative tissues

Unlike *etdf1*, pollen was visible by eye on the anthers of *erec8* flowers (Figure 5). However, the pollen staining protocol we used to study pollen grains in *erec8* plants indicated that almost all pollen grains produced were aborted, as indicated by a lack of magenta‐red color in spores, in addition to the reduced size and abnormal shape (not triangular) of aborted grains relative to non‐aborted grains (Figure 6, Table S13). Of note, a high rate of pollen abortion was also seen in control events with the pollen germination assay used here, although prior work with *FT‐*expressing hybrid Eucalyptus demonstrated high rates of fertility as measured by distinct seed germination assays (Klocko et al., 2016). This may be a consequence of reduced efficiency of pollen germination in vitro, partial loss of viability during storage of pollen at 4°C for up to 1 month, the many generations of clonal propagation since the earlier work was done, and/or changes in greenhouse conditions over time. It is also likely contributed to by the interspecific hybrid nature of the Eucalyptus line being studied.

To follow up on pollen viability staining, we performed a germination assay and found that no pollen germination occurred in *erec8* samples, whereas most control plants displayed some level of germination (Table S15; Fisher's exact test one‐sided *p*‐value = .013). Given that notable rates of pollen abortion were observed in control lines in our experimental settings with the pollen germination assay used, further research may benefit from the use of field studies and seed germination assays to provide more conclusive insights into pollen viability. Showing a trend similar to our results, staining of pollen from *REC8*‐RNAi knockdown rice indicated a predominance of aborted pollen, while pollen abortion was rare in control lines (Zhang et al., 2006). While not all pollen grains were aborted with *REC8‐*RNAi, this is likely because of the potential for some level of *REC8* function to persist with the use of RNAi‐mediated knockdown as opposed to CRISPR/Cas9‐mediated knockout, which appears to yield complete or near‐complete infertility in our case. To this end, knockout of the *REC8* homolog in rice (via radiation mutagenesis) has been reported to produce infertile plants with a complete lack of viable pollen as well as an inability to produce seeds when pollinated with wild‐type pollen (Shao et al., 2011). Complete male‐ and female‐sterility has also been reported in Arabidopsis loss‐of‐function *rec8* (Bai et al., 1999; Cai et al., 2003). Moreover, knockout of *REC8* via CRISPR/Cas9 in watermelon produced plants with inviable pollen and an inability to be fertilized by wildtype pollen, indicating both male‐ and female‐sterility (Cao et al., 2022).

We were unable to successfully complete pollination and seed production tests due to difficulties in obtaining developing fruit capsules under our greenhouse conditions, an effect that (as discussed above) may have been exacerbated by the hybrid nature of the eucalypt clone studied. However, considering the conserved mechanisms of *REC8* and other members of the cohesin complex in mediating chromosome condensation and pairing during male/female meiosis, together with the bisexual infertility of loss‐of‐function mutants in Arabidopsis, rice, and watermelon, there is a high likelihood that our *erec8* Eucalyptus will be female‐ as well as male‐sterile. To this end, our early‐stage findings encourage further research to clarify the mechanism and extent of female‐ and male‐sterility in *erec8* plants, including fluorescent DNA staining and microscopy to inform whether sterility results from defective meiosis as with loss‐of‐function of Arabidopsis (Bai et al., 1999), rice (Shao et al., 2011), and watermelon (Cao et al., 2022) homologs. In addition, field trials would provide opportunities for *in planta* pollination assays, seed germination assays, and empirical confirmation of female‐ and male‐sterility.

Published transcriptome data for *E. grandis* indicates that expression of *EREC8* is upregulated in flowers, but expression is also present in vegetative tissues (Table S14; Vining et al., 2015); in contrast, expression of *REC8* in Arabidopsis may be almost entirely specific to flowers, although with low levels of expression in seeds and roots (Figure S11; Klepikova et al., 2016). In rice, *REC8* expression was detected not only in flowers but also at low levels in multiple vegetative tissues, more similarly to Eucalyptus (Zhang et al., 2006). Studies on knockdown and knockout phenotypes in Arabidopsis, rice, and watermelon similarly lack any report of vegetative alterations (Bai et al., 1999; Cai et al., 2003; Cao et al., 2022; Shao et al., 2011; Zhang et al., 2006). Vegetative expression does not necessarily imply a functional role in the development of any somatic tissue; indeed, mitosis utilizes functionally distinct homologs of *REC8* in the mitotic cohesion complex rather than *REC8* itself (da Costa‐Nunes et al., 2006).

To assess the potential ecological function of *erec8* mutant flowers, we performed a rapid and informal assay of sugar content in their nectar and that of *FT‐*only controls. Approximate sugar content, measured with a Brix refractometer, appeared to be similar regardless of whether *EREC8* was knocked out (Table S16), suggesting that although *erec8* pollen was inviable, the nectar may still provide nutritional benefits to ecologically valuable pollinators that rely on nectar for nutrition. Many species of *Eucalyptus*, across many localities, are pollinated by diverse insects and birds, both in the wild and in agroforestry systems (Bezemer et al., 2016, 2019; Hingston et al., 2004; Ottewell et al., 2009). We therefore speculate that the use of *erec8* for genetic containment, as opposed to fully pollenless plants (e.g., *etdf1* and *ehec3‐like*) or those without flowers at all (e.g., *eleafy* mutants; Elorriaga et al., 2021), would help to support many pollinator species. Field research is, of course, required to evaluate whether these potential benefits are detectable or substantial.

### 3.6 *EHEC3‐like* is necessary for pollen and stigma development, differing from the Arabidopsis homolog

**FIGURE 7:**
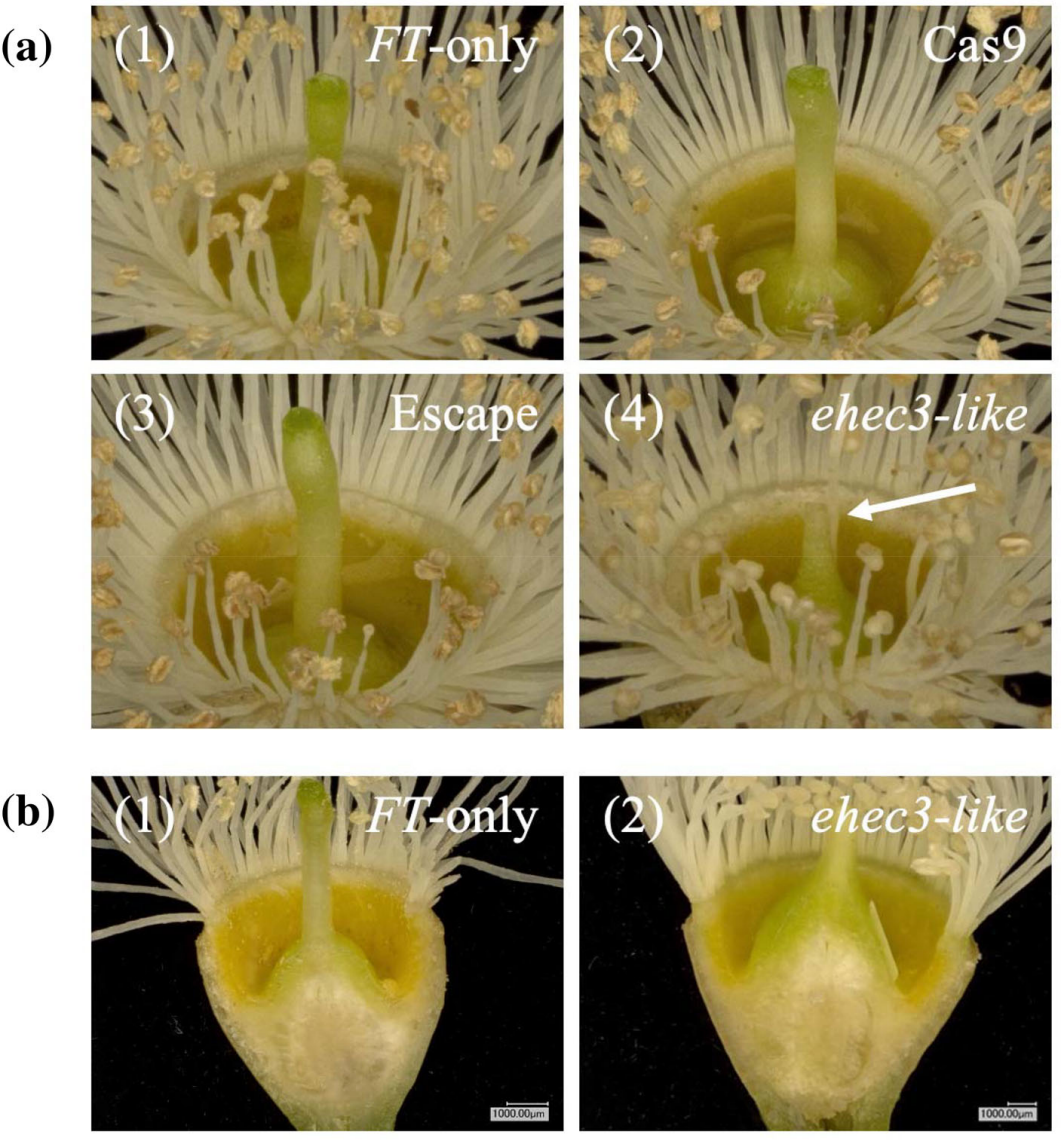
Style length was reduced and the stigma appeared absent in *ehec3‐like* flowers. (a) Floral images showing the style of three types of controls (*FT*‐only, Cas9, escape; Figure 1), and *ehec3‐like*, respectively [1: ramet 35, 2: ramet 124, 3: ramet 132, 4: ramet 30]. In panel (a)‐(4), an arrow points to the short style with an absence of an obvious visible stigma in the *ehec3‐like* flower. (b) Cross‐sections of gynoecium in *FT*‐only control (1) and short‐styled *ehec3‐like* flowers (2) [1: ramet 107, 2: ramet 60].

Knockout of our final candidate gene, *EHEC3‐like*, yielded phenotypes affecting both male and female organs and fertility. Carpels were absent of an obvious well‐formed stigma (Figure 7) and had abnormally short styles (*p* = .02, one‐tailed Student's *t*‐test; Figure 7). We also identified a male phenotype of failed pollen development, recognizable by the eye as an absence of pollen on anthers (Figure 5) and further supported by a lack of pollen found by attempted collection and microscopy of pollen grains (Figure 6, Table S13). Of note, a single *ehec3‐like* ramet from a single event studied appeared to produce a very small number of pollen grains. This could easily be because of sample contamination during pollen handling, some residual chimerism in the transgenic plants (where not all cells have loss‐of‐function alleles), or suggest that male‐sterility is not absolute in all knockout mutations of this gene.

While *HEC3* and both other *HEC* family genes of Arabidopsis have functional roles in carpel development, knockout phenotypes indicate that the specific roles are functionally divergent to some extent, despite overlapping in function. In Arabidopsis, stigmas continue to develop despite the loss of functional *HEC3* unless combined with additional loss‐of‐function or RNAi suppression with respect to *HEC1*, *HEC2*, and *HEC3* simultaneously, indicating functional redundancy. Simultaneous knockout of *HEC1* and *HEC3* (*hec1*, *3)* led only to a reduction in stigma size and a reduction in fertility (Gremski et al., 2007). These findings from Arabidopsis contrast with our findings in *ehec3‐like* Eucalyptus lines, which only had a single gene knocked out but were absent of obvious visible stigmas. Moreover, our report of reduced style length in *ehec3‐like* eucalypts is contrary to reported knockout phenotypes in Arabidopsis, where *hec1*, *3* plants have slightly longer styles than wildtype, and RNAi‐mediated knockdown of *HEC2* in addition leads to further increased length (Gremski et al., 2007). Strikingly, we noted a complete or near‐complete absence of pollen from *ehec3‐like* eucalypts, even though the *HEC* family genes appear to have little or no expression in the stamens of Arabidopsis (Klepikova et al., 2016). Although we were unable to evaluate fruit and seed development in Eucalyptus, the combined knockdown or knockout of all three *HEC* family genes in Arabidopsis produces complete female infertility, likely due to the absence of stigmas (Gremski et al., 2007, Schuster et al., 2015). These results and prior reports suggest conservation of *HEC3* function only with respect to stigma development, with functional divergence in other tissues including the stamen and style, as well as a possible lack of redundancy among Eucalyptus *HEC* family genes. The comparable, although distinct, roles of *HEC3* across Eucalyptus and Arabidopsis, along with the failure of stigma to develop in *ehec3‐like* knockouts, suggest these knockouts are likely to be female‐ as well as male‐sterile.

In Arabidopsis, *HEC* family transcripts are detected in germinated seedlings and carpal tissues (Klepikova et al., 2016), including the septum and apical tips, followed by the stigma derived from the apical tips and the transmitting tract of the style (Gremski et al., 2007). Eucalyptus transcriptome data indicates that *EHEC3‐like* expression begins in early flowers prior to the maturation of carpals and stamen and increases substantially during late floral development when these reproductive tissues are well‐defined (Table S14; Vining et al., 2015). However, we lack more specific and comprehensive expression data to inform whether *EHEC3‐like* expression is also found in seedlings or the expression patterning in specific floral tissues such as stamens and carpels. Mechanistic research in Arabidopsis suggests that the roles of *HEC* family genes in gynoecium development depend on upstream and downstream auxin and cytokinin signaling, likely mediated at least in part by protein–protein interactions with the transcription factor SPATULA and transcriptional regulation by the auxin–responsive transcription factor ETTIN (Gremski et al., 2007; Schuster et al., 2014, 2015).

Among the three candidate genes we studied, *EHEC3‐like* appears to have the most complex relationship to homologs studied in other species. We speculate that *HEC* family genes and related genes (e.g., *IND1/EDA33*; Figure 3b) may be particularly subject to variation in reproductive function across species. To this end, *IND1* loss‐of‐function is reported to produce male‐sterility in *Brassica napus* L. (canola; El‐Mezawy et al., 2016) but not Arabidopsis (Kay et al., 2013). To better understand the distinct role of *EHEC3‐like* in floral development and how this role diverges from Arabidopsis *HEC3*, further work is needed to understand the expression patterns of *EHEC3‐like* in male and female floral tissues and the genetic regulatory networks through which this putative transcription factor acts. This work may utilize *in situ* hybridization to inform whether expression in stigma and precursor tissues is similar to that of Arabidopsis *HEC3* (Gremski et al., 2007) and to trace the absence of pollen in *ehec3‐like* to expression patterning in specific stages and tissues within stamens. Moreover, ChIP‐Seq may be useful to identify transcriptional targets of *EHEC3‐like*, followed by epistasis mutant experiments to elucidate the downstream pathway through which *EHEC3‐like* acts.

## 4 CONCLUSIONS

To study the effects of the three candidate genes on fertility and vegetative development, we constructed CRISPR/Cas9 constructs and used these to transform rapid flowering and normal (non‐flowering) genotypes of a *E. grandis × urophylla* hybrid. We demonstrated that *ETDF1*, *EREC8*, and *EHEC3‐like* are each necessary for pollen development and that male‐sterility is achieved by their knockout. Moreover, female‐sterility is also likely to result from the failure of stigma to develop in *ehec3‐like* mutants and from a failure in meiosis to yield female gametophytes in *erec8* mutants. We were unable to detect clear effects of knockouts in any gene on vegetative development. Our results suggest that knockouts of these genes are likely to be useful means to impart male‐ and/or female‐sterility in Eucalyptus without significant vegetative impact.

## ACCESSION NUMBERS

The accession numbers for the three Eucalyptus genes studied in this work are Eucgr.I02017 (*ETDF1*), Eucgr.G03083 (*EREC8*), and Eucgr.H04946 (*EHEC3‐like*). Reference sequences were obtained from the published *E. grandis* genome (Myburg et al., 2014), accessed via Phytozome (https://phytozome-next.jgi.doe.gov/).

## AUTHOR CONTRIBUTIONS

Steven H. Strauss, Amy L. Klocko, Michael F. Nagle, and Estefania Elorriaga designed the research. Candidate genes were selected by Michael F. Nagle, Amy L. Klocko, and Steven H. Strauss. Molecular cloning and bioinformatic analyses were performed by Michael F. Nagle, with guidance from Amy L. Klocko and Steven H. Strauss. Cathleen Ma led on plant transformation and tissue culture. Genotyping of mutants by PCR, submission of samples for Sanger sequencing, and evaluation of mutations was performed by Surbhi S. Nahata, Bahiya Zahl, Estefania Elorriaga, Greg S. Goralogia, Michael Gordon, and Michael F. Nagle. Plant propagation and caretaking were performed by Surbhi S. Nahata, Bahiya Zahl and Cathleen Ma. Vegetative phenotypes were measured by Surbhi S. Nahata and Bahiya Zahl, with statistical analyses performed by Michael F. Nagle. Floral phenotypes were collected and analyzed by Xavier V. Tacker, Alexa Niño de Rivera, Sonali Joshi, and Estefania Elorriaga. Results were interpreted by Michael F. Nagle and Steven H. Strauss, with input from other authors. Surbhi S. Nahata, Bahiya Zahl, Alexa Niño de Rivera, and Xavier V. Tacker wrote sections for the methods they performed, with revisions from Michael F. Nagle. Michael F. Nagle wrote the remaining sections, with support from Steven H. Strauss. All authors contributed revisions.

## CONFLICT OF INTEREST STATEMENT

The authors report no conflicts of interest regarding this work.

### PEER REVIEW

The peer review history for this article is available in the Supporting Information for this article.

## Supporting information


**Data S1.** Supporting Information.

**Data S2.** Supporting Information.

**Table S1.** **Target sites for CRISPR/Cas9‐mediated mutagenesis of each target gene.** Candidate sgRNA sites were identified using CRISPRDirect based on PAM sequences, GC content, and possible off‐target sites, then ranked by predicted activity using sgRNAscorer v2.0.

**Table S2.** Primers for sequencing both alleles of *E. grandis* x urophylla hybrid and genotyping CRISPR/Cas9‐induced mutations of target genes.

**Table S3(A).** Specific mutations of each allele in each sample, as determined by sequencing in *etdf1* mutants with FT background.
**Table S3(B).** Specific mutations of each allele in each sample, as determined by sequencing in *erec8* mutants with FT background.
**Table S3(C).** Specific mutations of each allele in each sample, as determined by sequencing in *ehec3‐like* mutants with FT background.
**Table S3(D).** Specific mutations of each allele in each sample, as determined by sequencing in *etdf1* mutants with non‐FT background.
**Table S3(E).** Specific mutations of each allele in each sample, as determined by sequencing in *erec8* mutants with non‐FT background.
**Table S3(F).** Specific mutations of each allele in each sample, as determined by sequencing in *ehec3‐like* mutants with non‐FT background.

**Table S4.** Sample sizes of sequence‐confirmed knockouts and control plants surviving greenhouse acclimation.

**Table S5.** (A): Smith‐Waterman alignment results from alignment of *E. grandis* TDF1 (Eucgr.I02017) predicted peptide against predicted peptides from *A. thaliana* genome.
**Table S5 (B).** Smith‐Waterman alignment results from alignment of *E. grandis* HEC3‐Like (Eucgr.H04946) predicted peptide against predicted peptides from *A. thaliana* genome.

**Table S6.** (A): BLASTp results from alignment of *E. grandis* ETDF1 (Eucgr.I02017) against *A. thaliana* genome.
**Table S6 (B).** BLASTp results from alignment of *E. grandis* EREC8 (Eucgr.G03083) against *A. thaliana* genome.
**Table S6 (C).** BLASTp results from alignment of *E. grandis* EHEC3‐Like (Eucgr.H04946) against *A. thaliana* genome.

**Table S7.** (A): BLASTp results from alignment of *E. grandis* ETDF1 (Eucgr.I02017) against predicted peptides from the *E. grandis* genome and transcriptome for each hit. On heat map, red = low, yellow = intermediate, green = high expression.
**Table S7 (B).** BLASTp results from alignment of *E. grandis* EREC8 (Eucgr.G03083) against *E. grandis* genome and transcriptome data for each hit. On heat map, red = low, yellow = intermediate, green = high expression.
**Table S7 (C).** BLASTp results from alignment of *E. grandis* EHEC3‐Like (Eucgr.H04946) against *E. grandis* genome and transcriptome data for each hit. On heat map, red = low, yellow = intermediate, green = high expression.

**Table S8.** (A): Smith‐Waterman alignment results from alignment of *E. grandis* ETDF1 (Eucgr.I02017) predicted peptide sequence against predicted peptides from the *E. grandis* genome and transcriptome for each hit. On heat map, red = low, yellow = intermediate, green = high expression.
**Table S8 (B).** Smith‐Waterman alignment results from alignment of *E. grandis* EHEC3‐Like (Eucgr.H04946) predicted peptide against predicted peptides from *E. grandis* genome and transcriptome data for each hit. On heat map, red = low, yellow = intermediate, green = high expression.

**Table S9 (A).** Summaries statistics for various types of mutations.
**Table S9 (B).** Total numbers of observed mutations for each construct and background.
**Table S9 (C).** Total numbers of events, alleles and possible mutation sites used for computing summary statistics.

**Table S10.** (A): F‐test results for group of plants including *etdf1* and *ehec3‐like* events; without removal of any “escape” events.
**Table S10 (B).** F‐test results for group of plants including *erec8* events; without removal of any “escape” events.
**Table S10 (C).** EMM results for group of plants including *etdf1* and *ehec3‐like* events; without removal of any “escape” events.
**Table S10 (D):** EMM results for group of plants including *erec8* events; without removal of any “escape” events.

**Table S11.** (A): F‐test results for group of plants including *etdf1* and *ehec3‐like* events; with all “escape” events removed.
**Table S11 (B).** EMM results for group of plants including *etdf1* and *ehec3‐like* events; with all “escape” events removed.

**Table S12.** (A): F‐test results for group of plants including *erec8* events; with removal of “escape” event WT‐12.
**Table S12 (B).** EMM results for group of plants including *erec8* events; with removal of “escape” event WT‐12.

**Table S13.** **Mean pollen viability based on viability staining for control and knock‐out events.** Each row represents a sample of 1‐3 ramets of a given event, each with 2 buds collected. Total pollen per bud and viable pollen was approximated based on total and viable pollen per μL (as observed by viability staining and microscopy) and total sample volume. For events represented by a single ramet (and thus a single observation) the “average” computation includes a single value only, and is thus marked with a star (*) and includes no value for standard error (NA).

**Table S14.** Gene expression for three gene targets in *E. grandis* (from Vining et al. 2015).

**Table S15.** **Rate of pollen germination in *erec8* vs. control plants.** Pollen germination was assessed for eight pollen samples (each row is a single tree or ramet) each from the *FT‐*only control and from *erec8* knockout lines. Four *erec8* knockout events were studied, with each composed of pollen from two capsules of a given tree. Pollen germination was determined by performing our pollen germination assay (Materials and Methods) followed by examining pollen samples under 100x magnification and scoring them using the scale shown in Figure S6.

**Table S16.** Brix readings and approximate sucrose concentrations for nectar from *erec8* and FT‐only plants. Sucrose‐equivalent concentrations (final column) were computed using a standard curve for sucrose.

**Figure S1.** **Functional domains of peptide sequences in relation to sgRNA sites in three gene targets and their Arabidopsis homologs.** SMART predicted SANT‐Myb (SANT) and basic helix‐loop‐helix DNA‐binding domains as well as low‐complexity regions (purple) are shown for TDF1 and HEC3 peptide homologs in Arabidopsis and Eucalyptus. REC8 peptide functional domains (on the N‐terminus and C‐terminus) were not identified by SMART and were instead found in the Phytozome database, followed by producing a merged figure using information from both SMART and Phytozome. The approximate locations of sgRNA sites (Table S1) respective to the translated sequences are shown with scissors.
**Figure S2.** *EHEC3‐Like* gene sequence – Bold letters represent variants found in the *E. urophylla* sequence, while a yellow highlighted base represents a single‐base deletion found in the *E. grandis* allele that is not in the *E. grandis* reference genome. Blue highlighted letters represent guide RNA sites on the gene and green highlighted letters represent their PAM sites.
**Figure S3.** E*TDF1* gene sequence – Bold letters represent variants found in the *E. urophylla* sequence, while a yellow highlighted base represents a variant found in the *E. grandis* allele that is not in the *E. grandis* reference genome. Blue highlighted letters represent guide RNA sites on the gene and green highlighted letters represent their PAM sites.
**Figure S4.** *EREC8* gene sequence – Bold letters represent variants found in the *E. urophylla* sequence. Blue highlighted letters represent guide RNA sites on the gene and green highlighted letters represent their PAM sites.
**Figure S5.** Plasmid map for construct with two *ETDF1* gRNAs and kanamycin‐selectable marker. This figure was prepared in SnapGene and shows key features for the TDNA‐integrated region. Cloning was performed as described as described in Methods and Materials, and Jacobs & Martin, 2016. All transformation constructs follow this format, with substitutions for sgRNAs and selectable markers as described.
**Figure S6.** Scoring system for pollen germination. The extent of germination of pollen in a given sample was scored on a scale of 0 (no visible germination) to 3 (extensive germination), with examples for each score shown in this figure. All samples were observed under 100x magnification.
**Figure S7.** Residual plots for final volume index before log transformation. Scedasticity was assessed with scattergrams of standardized residuals against fitted values (top) and with Q‐Q plots of residual values against a theoretical normal distribution with the same mean and variance (bottom). (A) Standardized residuals vs. fitted values for the group of plants with *etdf1*, *ehec3‐like* and controls. (B) Standardized vs. fitted values for the group of plants with e*rec8* and controls. (C) Normal Q‐Q plot of the group of plants with *etdf1*, *ehec3‐like* and controls. (D) Normal Q‐Q plot of the group of plants with e*rec8* and controls.
**Figure S8.** Residual plots for leaf mass before log transformation. Scedasticity was assessed with scattergrams of standardized residuals against fitted values (top) and with Q‐Q plots of residual values against a theoretical normal distribution with the same mean and variance (bottom). (A) Standardized residuals vs. fitted values for the group of plants with *etdf1*, *ehec3‐like* and controls. (B) Standardized vs. fitted values for the group of plants with e*rec8* and controls. (C) Normal Q‐Q plot of the group of plants with *etdf1*, *ehec3‐like* and controls. (D) Normal Q‐Q plot of the group of plants with e*rec8* and controls.
**Figure S9.** Residual plots for volume index after log transformation. Scedasticity was assessed with scattergrams of standardized residuals against fitted values (top) and with Q‐Q plots of residual values against a theoretical normal distribution with the same mean and variance (bottom). (A) Standardized residuals vs. fitted values for the group of plants with *etdf1*, *ehec3‐like* and controls. (B) Standardized vs. fitted values for the group of plants with *e* *rec8* and controls. (C) Normal Q‐Q plot of the group of plants with *etdf1*, *ehec3‐like* and controls. (D) Normal Q‐Q plot of the group of plants with e*rec8* and controls.
**Figure S10.** Residual plots for leaf mass after log transformation. Scedasticity was assessed with scattergrams of standardized residuals against fitted values (top) and with Q‐Q plots of residual values against a theoretical normal distribution with the same mean and variance. (A) Standardized residuals vs. fitted values for the group of plants with *etdf1*, *ehec3‐like* and controls. (B) Standardized vs. fitted values for the group of plants with e*rec8* and controls. (C) Normal Q‐Q plot of the group of plants with *etdf1*, *ehec3‐like* and controls. (D) Normal Q‐Q plot of the group of plants with e*rec8* and controls.
**Figure S11.** Expression of *AtREC8*, also known as *AtSYN1*. This figure was produced using the Arabidopsis eFP Browser (bar.utoronto.ca).
**Figure S12.** Expression of *AtSYN2*. This figure was produced using the Arabidopsis eFP Browser (bar.utoronto.ca).
**Figure S13.** Expression of *AtSYN3*. This figure was produced using the Arabidopsis eFP Browser (bar.utoronto.ca).
**Figure S14.** Expression of *AtSYN4*. This figure was produced using the Arabidopsis eFP Browser (bar.utoronto.ca).
**Figure S15.** Correlations between traits helped to differentiate outliers. Plots allow visualization of distinct or extreme values and their relationships across traits. Traits underwent a Z‐score transformation prior to plotting to bring them onto a similar scale for easy visualization. This plot was produced with `ggpairs` from the GGAlly package in R. Cas9 control events are divided between event #7‐1, which has eight ramets with distinct values (red), and all other Cas9 control events (green), and thus was excluded from final analyses. A single ramet from the “escape” control group displayed distinct values for leaf mass and leaf area (WT‐12) and was thus also excluded from statistical analyses.
**Figure S16.** Relationships between initial volume index and traits of interest. Data shown is from the *etdf1* and *ehec3‐like* experimental group, which included control escape (Ctl. Esc.) plants that were transferred to the greenhouse at a different time than experimental lines and were thus of a different size.

## Data Availability

All data is available upon request to the corresponding authors.
