# Supplementary material for: Knockout of floral and meiosis genes using CRISPR/Cas9 produces male‐sterility in Eucalyptus without impacts on vegetative growth
Source: Plant Direct. 2023 Jul 14;7(7):e507. doi: 10.1002/pld3.507 (PMC10345981; doi:10.1002/pld3.507)
Supplement: Supplementary file 1 — Data S1. Supporting Information. [file PLD3-7-e507-s008.docx]

**Supplemental Results**

*Handling of outliers and aberrant data*

Our F-test and EMM analyses were performed first using all datapoints except those for the unusual event 7-1 of plants transformed with the Cas9 control vector in the batch featuring *erec8* plants. This event was represented by eight ramets, out of a total 87 ramets studied for each of 10 Cas9 control events (Fig. S15). These eight ramets tended to display extreme values for volume index (low) and chlorophyll content (high) as well as for oil gland density (low). Considering this, we speculated that the transgene insertion for this event may have knocked out or significantly altered expression of a gene regulating general development. Thus, the eight ramets for this event were excluded from further analysis.

Our initial F-test results indicated that there was no significant effect of transformation construct in the batch of plants that included *erec8* mutants, but there was a statistically significant effect (*p* = 0.0103) in the batch of plants including *tdf1* and e*hec3-like* mutants for one trait: leaf mass. Initial EMM results also supported an effect on leaf mass in this batch (*p* = 0.0373), but indicated that this effect only appeared when comparing “escape” control plants to *ehec3-like* mutants (Table S10). We note that for the *etdf1* and *ehec3-like* batch, the “escape” controls were rooted approximately two weeks prior to the other plants and were of a larger initial size upon potting and transfer to the greenhouse. Considered together with the correlation between initial size and leaf mass at the time of data collection (Fig. S16), this observation suggests that these controls were subject to a confounding influence, and we therefore excluded them from a second round of statistical analysis (Table S11). Additionally, we noted that the “escape” control event WT-12, used in the *erec8* batch, displayed outlier characteristics (Fig. S15) and thus excluded it from a second round of analysis for this batch (Table S12). Neither of these two models constructed after initial tests displayed any significant effects at α = 0.05, although F-tests and EMM tests may both suggest the possibility of effects on leaf mass and oil gland count at α = 0.10 in the *etdf1/ehec3-like* group. This result suggests that a larger trial with more statistical power may be able to detect a significant effect. F-test results from selected models are presented for each batch are summarized in Table 1, including the initial model for the *erec8* batch (detailed in Table S10.B) and the second model for the *etdf1/ehec3-like* batch excluding “escape” controls (detailed in Table S11.A).
