# Supplementary material for: Knockout of floral and meiosis genes using CRISPR/Cas9 produces male‐sterility in Eucalyptus without impacts on vegetative growth
Source: Plant Direct. 2023 Jul 14;7(7):e507. doi: 10.1002/pld3.507 (PMC10345981; doi:10.1002/pld3.507)
Supplement: Supplementary file 3 — Table S1. Target sites for CRISPR/Cas9‐mediated mutagenesis of each target gene. Candidate sgRNA sites were identified using CRISPRDirect based on PAM sequences, GC content, and possible off‐target sites, then ranked by predicted activity using sgRNAscorer v2.0. [file PLD3-7-e507-s001.docx]

| **Gene** | **Position in genomic DNA sequence** | | | **Sequence and PAM** | **GC content** | **Genome-wide PAM-proximal matches** | | | **Activity** |
| --- | --- | --- | --- | --- | --- | --- | --- | --- | --- |
|  | *Start* | *End* | *Strand* |  |  | *20-mer* | *12-mer* | *8- mer* |  |
| *ETDF1* | 45 | 67 | - | CCTTTGGACGCCCGAAGAAGATG | 55% | 1 | 1 | 491 | 65.055 |
| *ETDF1* | 912 | 934 | - | CCCACAAGCCTTTCTCTCAGATC | 45% | 1 | 1 | 1184 | 88.410 |
| *EREC8* | 3898 | 3920 | - | CCCCATGACTCACCTTCTGGTCA | 50% | 1 | 1 | 1408 | 93.006 |
| *EREC8* | 4169 | 4191 | + | GCCTTTCGAAGATCTACACGTGG | 50% | 1 | 1 | 255 | 94.348 |
| *EHEC3-Like* | 273 | 295 | + | GGAGATGATGTACAAGATTGCGG | 40% | 1 | 1 | 1472 | 86.444 |
| *EHEC3-Like* | 425 | 447 | + | TCCTCCAGAGGCTCGTCCCCGGG | 70% | 1 | 1 | 318 | 67.120 |

**Supplemental Table 1. Target sites for CRISPR/Cas9-mediated mutagenesis of each target gene.** Candidate sgRNA sites were identified using CRISPRDirect based on PAM sequences, GC content, and possible off-target sites, then ranked by predicted activity using sgRNAscorer v2.0.
