# Supplementary material for: Knockout of floral and meiosis genes using CRISPR/Cas9 produces male‐sterility in Eucalyptus without impacts on vegetative growth
Source: Plant Direct. 2023 Jul 14;7(7):e507. doi: 10.1002/pld3.507 (PMC10345981; doi:10.1002/pld3.507)
Supplement: Supplementary file 15 — Table S13. Mean pollen viability based on viability staining for control and knock‐out events. Each row represents a sample of 1‐3 ramets of a given event, each with 2 buds collected. Total pollen per bud and viable pollen was approximated based on total and viable pollen per μL (as observed by viability staining and microscopy) and total sample volume. For events represented by a single ramet (and thus a single observation) the “average” computation includes a single value only, and is thus marked with a star (*) and includes no value for standard error (NA). [file PLD3-7-e507-s002.docx]

| **Genotype** | ***FT* Line** | **CRISPR/Cas9 Event** | **Number of ramets sampled** | **Average % Viability** | **Approximate total pollen per bud** | |
| --- | --- | --- | --- | --- | --- | --- |
|  |  |  |  |  | **Average** | **Standard error** |
| *FT*-only control | 4-2 | NA | 2 | 14.52 | 24,507 | 9,909 |
|  | 30-3 | NA | 2 | 7.97 | 1,034 | 365 |
| Cas9 control | 4-2 | 1-2 | 1 | 10.00 | 21,150* | NA |
|  | 30-3 | 5-1 | 2 | 26.30 | 1,022 | 192 |
|  | 30-3 | 15-2 | 2 | 17.20 | 29,955 | 3,680 |
|  | 30-3 | 13-3 | 1 | 6.40 | 1,863* | NA |
| Escape control | 4-2 | NA | 2 | 21.89 | 22,575 | 12,744 |
| *ehec3-like* | 4-2 | 16-1 | 3 | 0.00 | 0 | 0 |
|  | 30-3 | 9-1 | 1 | 50.00 | 25* | NA |
|  | 30-3 | 18-2 | 1 | 0.00 | 0* | NA |
|  | 30-3 | 3-1 | 1 | 0.00 | 0* | NA |
| *etdf1* | 30-3 | 6-1 | 2 | 0.00 | 0 | 0 |
|  | 30-3 | 28-1 | 3 | 0.00 | 0 | 0 |
|  | 30-3 | 5-1 | 1 | 0.00 | 0* | NA |
| *erec8* | 4-2 | 18-2 | 3 | 0.00 | 219 | 54 |
|  | 4-2 | 34-2 | 3 | 0.26 | 8,958 | 3,356 |
|  | 30-3 | 29-1 | 2 | 0.00 | 5,175 | 1,305 |
|  | 30-3 | 8-2 | 2 | 0.00 | 853 | 594 |
|  | 30-3 | 20-2 | 2 | 0.00 | 5,175 | 2,153 |

**Supplemental Table 13. Mean pollen viability based on viability staining for control and knock-out events.** Each row represents a sample of 1-3 ramets of a given event, each with 2 buds collected. Total pollen per bud and viable pollen was approximated based on total and viable pollen per μL (as observed by viability staining and microscopy) and total sample volume. For events represented by a single ramet (and thus a single observation) the “average” computation includes a single value only, and is thus marked with a star (*) and includes no value for standard error (NA).
