# Supplementary material for: Knockout of floral and meiosis genes using CRISPR/Cas9 produces male‐sterility in Eucalyptus without impacts on vegetative growth
Source: Plant Direct. 2023 Jul 14;7(7):e507. doi: 10.1002/pld3.507 (PMC10345981; doi:10.1002/pld3.507)
Supplement: Supplementary file 17 — Table S15. Rate of pollen germination in erec8 vs. control plants. Pollen germination was assessed for eight pollen samples (each row is a single tree or ramet) each from the FT‐only control and from erec8 knockout lines. Four erec8 knockout events were studied, with each composed of pollen from two capsules of a given tree. Pollen germination was determined by performing our pollen germination assay (Materials and Methods) followed by examining pollen samples under 100x magnification and scoring them using the scale shown in Figure S6. [file PLD3-7-e507-s019.docx]

| **Genotype** | **FT Event** | **CRISPR/Cas9 Event** | **Pollen Germination (on 0-3 scale)** |
| --- | --- | --- | --- |
| ***FT* control** | 30-3 | NA | 0 |
|  | 30-3 |  | 0 |
|  | 30-3 |  | 0 |
|  | 30-3 |  | 2 |
|  | 4-2 |  | 2 |
|  | 4-2 |  | 2 |
|  | 4-2 |  | 1 |
|  | 4-2 |  | 1 |
| ***erec8*** | 30-3 | 8-2 | 0 |
|  | 30-3 | 8-2 | 0 |
|  | 30-3 | 29-1 | 0 |
|  | 30-3 | 29-1 | 0 |
|  | 4-2 | 34-2 | 0 |
|  | 4-2 | 34-2 | 0 |
|  | 4-2 | 18-2 | 0 |
|  | 4-2 | 18-2 | 0 |

**Supplemental Table 15. Rate of pollen germination in *erec8* vs. control plants.** Pollen germination was assessed for eight pollen samples (each row is a single tree or ramet) each from the *FT-*only control and from *erec8* knockout lines. Four *erec8* knockout events were studied, with each composed of pollen from two capsules of a given tree. Pollen germination was determined by performing our pollen germination assay (Materials and Methods) followed by examining pollen samples under 100x magnification and scoring them using the scale shown in Fig. S6.
