# Supplementary material for: Knockout of floral and meiosis genes using CRISPR/Cas9 produces male‐sterility in Eucalyptus without impacts on vegetative growth
Source: Plant Direct. 2023 Jul 14;7(7):e507. doi: 10.1002/pld3.507 (PMC10345981; doi:10.1002/pld3.507)
Supplement: Supplementary file 19 — Figure S1. Functional domains of peptide sequences in relation to sgRNA sites in three gene targets and their Arabidopsis homologs. SMART predicted SANT‐Myb (SANT) and basic helix‐loop‐helix DNA‐binding domains as well as low‐complexity regions (purple) are shown for TDF1 and HEC3 peptide homologs in Arabidopsis and Eucalyptus. REC8 peptide functional domains (on the N‐terminus and C‐terminus) were not identified by SMART and were instead found in the Phytozome database, followed by producing a merged figure using information from both SMART and Phytozome. The approximate locations of sgRNA sites (Table S1) respective to the translated sequences are shown with scissors. Figure S2. EHEC3‐Like gene sequence – Bold letters represent variants found in the E. urophylla sequence, while a yellow highlighted base represents a single‐base deletion found in the E. grandis allele that is not in the E. grandis reference genome. Blue highlighted letters represent guide RNA sites on the gene and green highlighted letters represent their PAM sites. Figure S3. ETDF1 gene sequence – Bold letters represent variants found in the E. urophylla sequence, while a yellow highlighted base represents a variant found in the E. grandis allele that is not in the E. grandis reference genome. Blue highlighted letters represent guide RNA sites on the gene and green highlighted letters represent their PAM sites. Figure S4. EREC8 gene sequence – Bold letters represent variants found in the E. urophylla sequence. Blue highlighted letters represent guide RNA sites on the gene and green highlighted letters represent their PAM sites. Figure S5. Plasmid map for construct with two ETDF1 gRNAs and kanamycin‐selectable marker. This figure was prepared in SnapGene and shows key features for the TDNA‐integrated region. Cloning was performed as described as described in Methods and Materials, and Jacobs & Martin, 2016. All transformation constructs follow this format, with substitutions for sgRNAs and selectab [file PLD3-7-e507-s015.docx]

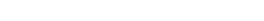


**
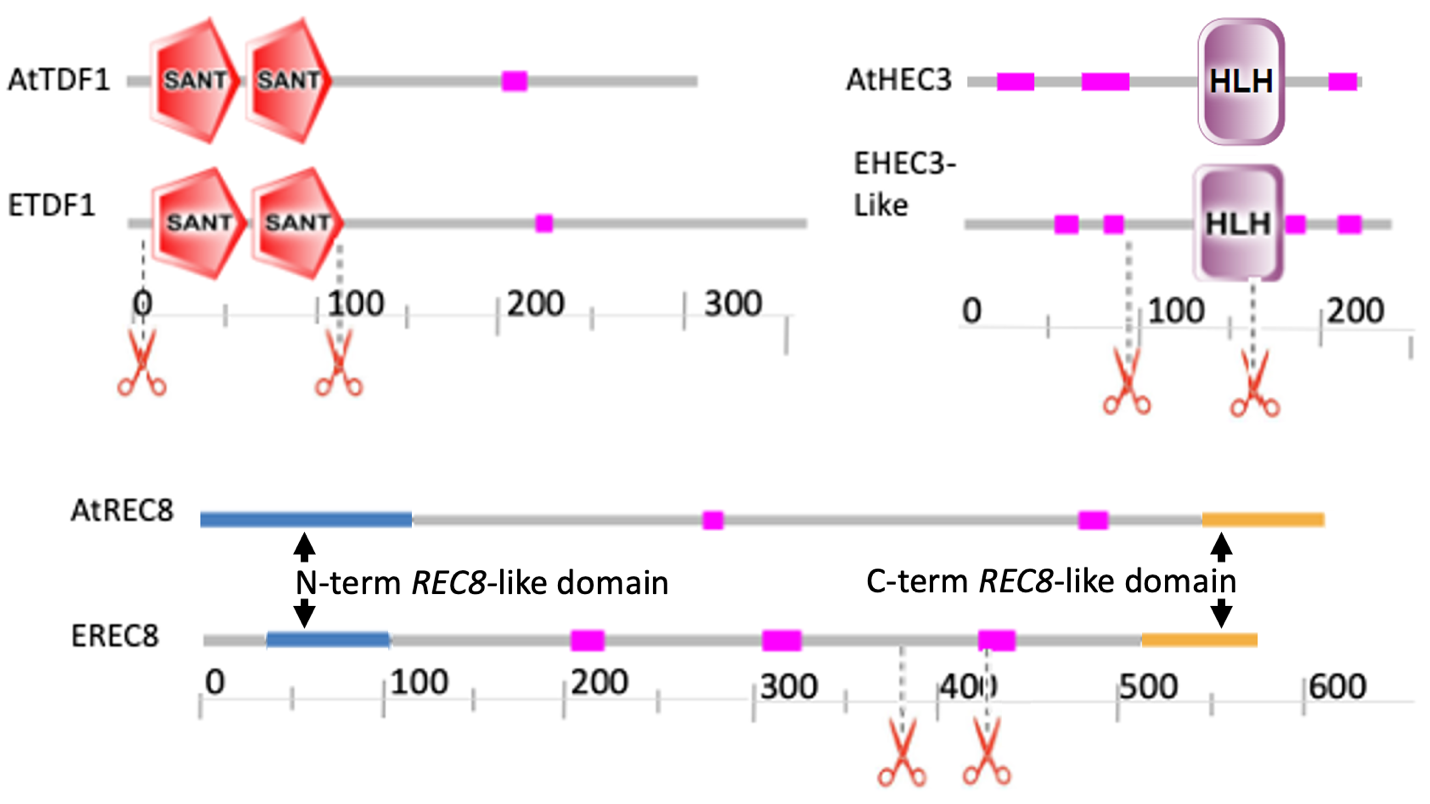
Supplemental Figure 1. Functional domains of peptide sequences in relation to sgRNA sites in three gene targets and their Arabidopsis homologs.** SMART predicted SANT-Myb (SANT) and basic helix-loop-helix DNA-binding domains as well as low-complexity regions (purple) are shown for TDF1 and HEC3 peptide homologs in Arabidopsis and Eucalyptus. REC8 peptide functional domains (on the N-terminus and C-terminus) were not identified by SMART and were instead found in the Phytozome database, followed by producing a merged figure using information from both SMART and Phytozome. The approximate locations of sgRNA sites (Table S1) respective to the translated sequences are shown with scissors.

**>Eucgr.H04946 | Chr08:68856013..68856717 – *EHEC3-Like* (Grandis reference sequence modified to feature SNPs identified by sequencing)**
ATGGACTTCAACCAAAGCAAGTTCTCGAACAACTTTTGGGATCTTGGCTTGGGCATGGAAGATCAAACCCTCCATTCCAATGACCAACATCATCATCAGCCGCCTTTCACTTCCCTTTGGCCTAGCATCCATCTCCCATTAATGCACCAAACAACTCCAACTACTTCCCAGATTCCATCTTCCCACTTTGTGAATGATAGTAGCATTGGGGTTGTGGCAAATCAAATCGAAGACAAGGATGAAGA(**A/**G)CCTGAAGAGGAGCTTGGAGCCATGAAGGAGATGATGTACAAGATTGCGG(**C/**T)GATGCAACC(**GG**/_A)T(**T/**C)GACGT(**T/**C)GACCCGACGACGATTCGGAAGCC(**A/**G)AAGAGGCGGAATGTCCGGATCAGTGATGATCCTCAAAGTGTAGCGGC(**A/**G)CGCCT(**T/**C)CGGCGGGAAAGGATAAGCGAGAAGATCAGGATCCTCCAGAGGCTCGTCCCCGGGGGGAC(**G/**A)AAGATGGACACGGCTTCGATGTTGGACGAGGCTATTCGCTATGTCAAGTTCTTGAAGCGACAAATCCGTTTGCTTCAACAGCCAAATAACCAAAACCCTACACCAGATCCGGGCGCAGTCACGGGGAGCTTGGTGGGAGGGGATTGGCAAGGCGTGATTGCACCGAGCAAACCACCCTCCACTACCTCGTCATCGTCCTTCGAAGCACAGGTCGGATCAAGGTTTGGGTTCTGGTCCAATGGTGGTGGTGGT

**Supplemental Figure 2.** ***EHEC3-Like* gene sequence** – Bold letters represent variants found in the *E. urophylla* sequence, while a yellow highlighted base represents a single-base deletion found in the *E. grandis* allele that is not in the *E*. *grandis* reference genome. Blue highlighted letters represent guide RNA sites on the gene and green highlighted letters represent their PAM sites.

**>Eucgr.I02017 | Chr09:29814591..29816209 – *ETDF1* (Grandis reference sequence modified to feature SNPs identified by sequencing)**

ATGAAGAGACCGCCATGCTGCGACAAGTCGAACGTGAAGAGAGGCCTTTGGACGCCCGAAGAAGATGCCAAGATACTCGCATACGTATCGACTTACGGGACTGGCAACTGGACTTTGGTCCCTGAGAAAGCTGGTCTTCTTTTATGTTTCCAATCCTTTCATGAACAAGATCATTGTCCCTTCTCAGCTCGTTATTTCCATGTTTTGATAGAGCTCAGTCTTACTTTAGGTGTGCCCGCGCGCGCACATGCAGGATTAAACAGATGTGGAAAGAGCTG(**C**/T)AGGCTTCGGTGGACCAATTACCTGAGGCCTGACCTTAAGCATGATGGCTTTTCCCCTGAAGAAGAAGATCTCATCAT(**C**/A**)**AACCTCCACAAAATCTTTGGCAGCAGGTCTCACTAGTTTGCTCATCATTTCTCTGTCGAATGGCGTTATTCTTTTGAATGTATAAAGCCGATAGAATTTACAAGGAACGGAGTTTATCATGCATTTTCATTAGATTGAATGAATGATTCAAATCGCTGCTCATTTTCCTGTCGAGTTCCCCTCCGAATTTCACGGAGAAGACTCTTCCGCGGATGAGTGGATTAATGCAGACAGAGGGTTGTAGTTTTAACTTCGTAGATC(**A/**T)TGAGTTTGGAAGGAAACGTTAATGAACTCAACAGTTGGTGGGGTAAAGTTCATTATCAACCCATCTTTCTGTCATGAAAGGTTTCGATGTGTGTAACTTAATTCCCATGCATGCAGCATTTACGTATTTTCTAAGTACTCC(**A**/T)CTCACGCTTTTATTGGTTA(**T**/C)ATGATATGGTCTTGTTGCAGATGGTCTCTAATTGCAAAACATCTGCCCGGAAGAACAGACAACGATGTCAAGAACTACTGGAACACCAAGCTCAAGAAGAAGCTCCAAAAGATGGGAATCGATCCTTTAACCCACAAGCCTTTCTCTCAGATCTTCTCAGACTTTGAGAACATGAGCGGCTGCCCAAACGCCAGACATCGCCAAATCTTGCCCGCGCCATCGTGTTTGACTCAGGTCCCTGCAGGCTCCAATTCTCACTTGGATGCGATCATGAAGCCCGTGATGGAGCAAGTTCATGAGAATTTCACTGCCGAAAATCACCTCTCTTGGTCTCAGTACCAGGTGGCGAACCAAGATGTTATGCAGCTGCAACCATATCAATGTGTATTGAGCGAGGTCACGTCTTCTTGTTCCTCGTCATCCTCTCCTACTCTGACACGATTTAACACGCAACAATCGGATGGTCCACCGCTTCCTCCTCCCTTTGTTGCCTGGACTGGCGATTCGATTTCGCATCAGCCATTTCCCACTGGCAATGTATTGCCGAAGCGCGAAGGAGACTTGCAGGATATAGTGTCTTCATCGTCCATCAACTGTGCGAGCGATATGGCAAAACAAGCGCTCCCCAACACCCCTGTCGGCACAATGGCTTGTAAAAACGAAGCCAAATGGGCCGAACCCGGAGATTCGTGCGAGGGAGCGCTAGAACTGGATTGCCATTTCGGAGACGGTTCGTACTCTTGTTTCGGCTCTTTCACGGACGCCATCTTGGACAAGGACAGCAAGATGAGGTCGGAGTTATTTCCAGAATTTATAGATGGACTTCTAGATTACTGA

**Supplemental Figure 3.** **E*TDF1* gene sequence** – Bold letters represent variants found in the *E. urophylla* sequence, while a yellow highlighted base represents a variant found in the *E. grandis* allele that is not in the *E*. *grandis* reference genome. Blue highlighted letters represent guide RNA sites on the gene and green highlighted letters represent their PAM sites.

**>Eucgr.G03083 | Chr07:51979397..51985095 reverse – EREC8 (Grandis reference sequence modified to feature SNPs identified by sequencing)**

ATTTCCGATGTGGCATTCTTTCTCTGTTTTACGTCAACTTCGATACACCTGATTCTCCCGCTACATACAGAGTGTCCTGAAATAGCGCGGTAACTCAGGCCGGCGAACGAATGACGCGATCACATAGTCTCAGTCTCTATGATTTTACTGAGAAAAAAGGCGTTCTGATTCTGTGATTTTGATGAATGTTTGGGTTGATGAAGGATGGCCGCGACTATGCATGCGAAAATAAACCGGAGGAAGCTTAACAAGCTCGACATCATCCAGATATGGTACGGACAGACATTTCTCGTACTTTCAGCGTGCCGATGTTGCATTCGTGGAGTCACTGAAGCGTCCGTTCTTCTTTTTACTCTTCAGCGAGGAGATCTTGAATCCGTCCATCCCGATGGCGCTCAGACTTTCCGGAATTCTGATGGGTATTTGAATTCCGCCCGTTGTTTTCTGGTTTCCTTCTTCATCCCGGTTTGCATGTTCCGTCGAATGGAACGCCTGATTCGTTATTGTCTCTCACAGGTGGAGTGGTGATTGTCTATGAGCGGAAAGTGAAACTCCTCTATGGTAAGCTTCCAGTTCCTCTCTTGCTATGGATTTCTTCATAATTTGAGAAAAAGAGAAATGAGTAACGCCGTTTCTTGATGTTGGGAATGATTTGCAGACGACGTCACTCGTTTCCTGGTACGATCATGACTCGATCAAAAACCCACACCCACCTCGACTTCACAAGCTTTTTTTTTCTATTTCTTATCCTTGCGTTCGTCTGTAGACCATGAATTTTTTGGTGGTATTAGGTTGAGATAAACGAGGCTTGGAAGGTTAAATCTGTTCCAGATCCAACGATCCTACCCAAGGGAAAAACTCAGGCCAAGTGAGTTGGGCTTGCTCCGCGTGATTTTTGTGCCGACCGGTTCCCTTCGATCTAGACTCGATTCATTTCATGTTTCGTAGGTGTAACCTAAAGAGTGTTTGTAGGATCGGCATTTAGTCCGAATCCGCATCTTTTGTGTCATTTTAGAAAGCTATAATTTCCCACAACGCCATTTTAATAGCCCAAAAAAAGATTAAGCAGAAACAGTGGGACGAGGGCAAGCTGGTCAAATTAATTTTGTTGAAGAGAGAAGTTCATGGCGTTTCTACATGTGTAGGAGAGAAGCGGTTACTCTGCCAGATAACCAGGAGACAGAGATCAGTGGTATCGAGCAACCCCTCCATTTCTCCTATGCCACGGCCACCATGGGTTTCCAACACACGGCGTACTTTGCTATGGTAATTTGCGCAATTTGATGTTCTTTGATCTTGTTGTGATATGTTGAGAATGCCTTTTGTATGTAGTGACTGCCGTTGATAGTACGATTTGGTGGTTCATTTTTTTCTTTTTGTGGCCATCTCATCTGATGATGATCTCAGAAAACCGTTTGGCATTTCCACTTCTGTACTGTCCTCATGACATCGCTCTCTCCGTGATTCGTTTGTGGTTGTACTATGGATATCATCTACAAGAGGATTTGGAGCGTACCGGATTGAAATGAAAAACATTATTATGATAGTTAAATTTGGTTTTCGGTTAAAACATCAGCAGAACTAGAATAGGGAAGGCAATAATTTGGTCGATATCGAGGGTAGGCGTTCTTGCAATAGCAGATATAGATAAAAATAGCGAGACATGTGTGACTTATTGGTATTTGAGTACGATAATGAGCCTGAGTGATCACCTTACAAATGGACTCTTCAACAGTCGTCAGTTTTGGAAATGCTAGCTAGTTAAGGCGATGATTCTAGAGAAAGGTAAATTCTTCACTAGCACAATCATCACATGCTTTATTGAAGAATTAGCAGGAAGATGAGTACATTGAACAATGGGAATGCCATTTATTGAATACTTCATTCTATGTTCAAGACTTGTCCAATGTTTGGATGATGATCTTTGTGAAAAGTAGCTTTAACTGTCATGTGCTTGAGTTTGACAGTGTTTGAGTTTCATATTTATAAATTAAGCAAGGATTCAAAGCTAGAGAAACACCTAAGCTAATGACAAATTCTTGAAGTTACCTGGCAACCTTAGGTATCGTTCACAAGAAAAGTGCACAACTTATATGTGGAATGTTGAATATGCCCAATTTCTCATGGCCATGGCTCTTCTGCTTAATTTTCGGTATTGCAGCGACTAGATAATGTTGACGAGACCTGCATGAATGACAATGAAAGGAATGTGGACCAAAATCAAAACTATCATCAAGGTTTGTGATCAACCCCATCATCTTTATCCAGCTTTTCCTCTTTTATCCGTATCGGTCAGATTAACATCATCAGTTCTCCATTTTCACAGCTGACGCAGCAAATATAACCTTATTCGATCATTTTGATCCTTATCAGGCTGATAGAGAACTATTTAATCACTTTGAAAGGTAATAAGAGCACTCTAACCTCAAAGGATGAGAACTCTAATATTAGATCTATATCTTTCTTTGGTCTACTTCTAAGAGAAAGGCTATCTTGTAATTGAGATGGAATATCTCGTTTCTAAACTTCACATTGTATTTGTGATGGAATATCCTGTTTGCTTAACACACCATCACAAGCATCTAGTTTTGCATTGCAATACCGTGTAATTGACAGGTTTGATATAGAGGGAGAGGACGAGACTCAGGCAAATTTTGCTTCAGGAGAGGATGCACACATGCCAAGTACTCTTGTACTCTCACCACCATTGCCTGATGATGCTCCGAGAGGTTTGCAGCACAATCACTTAAGTTTTCTGTTTACTGAATGGAGGCAAGTGTACATGAAGCTAGAACTGCTCGGCAATTATTTGATTTCTACTACCCGAGAACCAAATTTAGATCTATGTGAAAGAAACTTTACTTGACAGAGATCCTTCAGGGTGCACCACGTTGCCCATCCCCATATAGCAATTGATGAAGCAGTATATTCCTCTGAATTATTAAGTAGCTAATAGATTCATGATATGATCATATGAGTAGCTCTATGATTTTATCCTTAAGCATTTGACACAGTGATACGAACAGATTCATATTCATTTCCTTTAATTTCTGAGTCACGTCCGATAAAGCACTGAGCATGCATTGCAGATGGCCTTCGGTGTTAGAGACATATATTAAGATGCACACGGTTCTACTTGGATTTCTTTTCGAAATTAAAGTATTGAATTTTTAGCGAAAACACCTTACATAAATTGACTATTGCGCTTCCTGGCACAGAGGAAGTTCAAAATCAACATCCAGAAAGCCAAATAAATCAGGAAACTGATGATATCAAGTTTCCTCATCAGGTCACTAGTTAACTTACCAAAGTAACCTTTTCTCCTATAGAAGTAAAGACAATTTAGAGAGTTCACTGATAATGATAAAGGTGCGAATGAAGCAGGATAAGCAGAGACAGGGACAAGCCAAAAGGAAAGCTAGAAGAGCAGAACCTTTTGCCATGGACTACGAACAGACAATCATCCCTGGAAATATATACCAATTATGGCTGCAAAACTCTTCAAGCATTGTCTCAAAAAGAGGAAGAAAGAGAAAGGTTAGTGCTTCTCCTTTTGGGTCCAACGCAGGACTACATGGCCGAGTCAAGC(**C**/A)ATTTTTGTGAACTAACCTGTCCCTTCCGCTGGGTGCTTGGAGGGTTGGATGACTAAATTTGGAGGGACATCCATGTTAGGAGTTTCAGCTTTCAGCCTTTTATGTTAAACAGTAACAATAAAAAAACTC(**C**/T)GATGAGACAATTCCGAGATGCATTTTCTTGCAGCGTACATGTGTGATGAACAGCATGAAGATCGCTCATCTCATGGAGCTACCACCAATAGTACTAGCATGTGGTTTACTGAATGAAAATAGAAGCATCTATTATCCTCCTCCTCTCATGGAGCTGTGGACGAGAAGTATCCAGCCTCCCCATGACTCACCTTCTGGTCAGCCTTTGGCAAGCCTCTTTTATAGCAATTTGTCGTGCTTGATAGAGTGCAACCCCAGTTCAAAATAATAACCGTTCTCCACAGGAAGAACATCCCCGCCCTTGCCACCAGAACCATCATCGTCATCCCC(**A**/C)CCAGAGAGAGCATATTATCAAGATTTAGGGGCACGGGTATGCCTCTCATCTTTTTACTGGAGTACCTGATTGCAATGTGTGTGTCTGGACTCCGTCTGATGAA(**G**/A)TATTTGATTGTGAAGCCTTTCGAAGATCTACACGTGGAGGTCAACCCACA(**G**/T)TCACAAGATGTCTCCATAGAGAAGCTGCGGAATAATTTTGAAAATC(**A**/G)GGAAATGCCCCCGGAAATTTTCATGGAAGGATTTAGAAACAATCTCATCCACAACAAGACAGCGGCAGCAGATCCGGACCCACTGGTAACCCCAGGAAATTCAGGTAATGCTTACGTATTGGGATGTATCACATATTGCGGAGAACAAGCAAAAATCTCTTTGCATATGGTATTACAGGGGATGATGTAAGATCCATACCAAGCTCAGCTTCTGGACACAACTTCATAGCACACAATGTGGAAGTCAACTCCGGACGGTTAGTGAGAATATGCATCTTTCCTAAGAGTGCTTTGGATAGTGCACAAAGATTTACAGGCAGGAATCAAATTTTCTCATGAGAGCAAATTAATACTATCGTGGAACTAGTTGGTTCTATCTTGCATCTTATTAGTTTGTCTCATTTACTGAACAAATAATTGATCATATGGATCGACATCATTGTACCTAATAGATCCAGCAAGAAGCGGCCCTTTTCTTCATCTAGAAACAGCAGTAGTGGCCTCGAACCTGTAGCTGAGGAAAATTCATGGTTAAACGGGGGTCCCAACTTCAAGTTATCAAGGCTGCCTGAAGACGGCCAAACACCCGGTAGGTATCCCATTGATATCAAAGAGAAAGTGCACAGTCATGTCATATTGTCGATTACTTAGAGACGCACGGCAAGTGGGCGACCTTCTTAAGTGTTCTCTTTCTTGAGCGTCTCCTGCAGAACTGCTGGTGGAAACTGGACCAACACAAACCCAACGCCCAATCAGGAGTCCTCCTGTTGACAAGCTTACAGATACTATCCGCACGTATGTTGCAAAATCTTCTCAATCTATCTGCTCAAAGTTTACTTTTCCTTGGCATAATCAAGGTCTACCGTACCCTCTCTTAATGGCTGCAGCCAGATGAAAGAGCATTTTGAGACTCCCGGGGCCCCCAAGGAAGAATCCCTGAACCACCTCGCCACTGGGATGAGCCGAAAAGGAGCAGCCACGCTCTTCTATCAGACTTGCGGTACTTGTCCATGCAATGATCTAATCGCTTTCGGTCAATGAAACGCTCACGATTCACTGGCTTACAGCTTATTTCAAACCCGCAAGACCAAGATACCAAGATTAATCAGCTGATTTGTTGATGCAGTTCTTGCGACTCGTGATATGCTCGGAGTTGAACAAAGGGTGCCTTATGGAGATATACTCATTTCTCGAGGAGCGAAAATGTGATTCCACTTAAGGTCCACATTTCCCGCGTGAAAACTCTTTCTACTTTTCCTGCTCTTCTTGCCGGAACGTTATGCAAGAGCTAAACTGAGAAACCTTCTCCGTTTCCTACAGATTGAAACAAGGCGGACCTGAACATGTCCTAAATTTTTCTTCGGGACGACGGCCTATTTTGATGACAGTGGGCAACTCTTAGACTGAGCTTCTCTTTCTTGAAGTGCGATATCTATTTGATATTTCC

**Supplemental Figure 4.** ***EREC8* gene sequence** – Bold letters represent variants found in the *E. urophylla* sequence. Blue highlighted letters represent guide RNA sites on the gene and green highlighted letters represent their PAM sites.


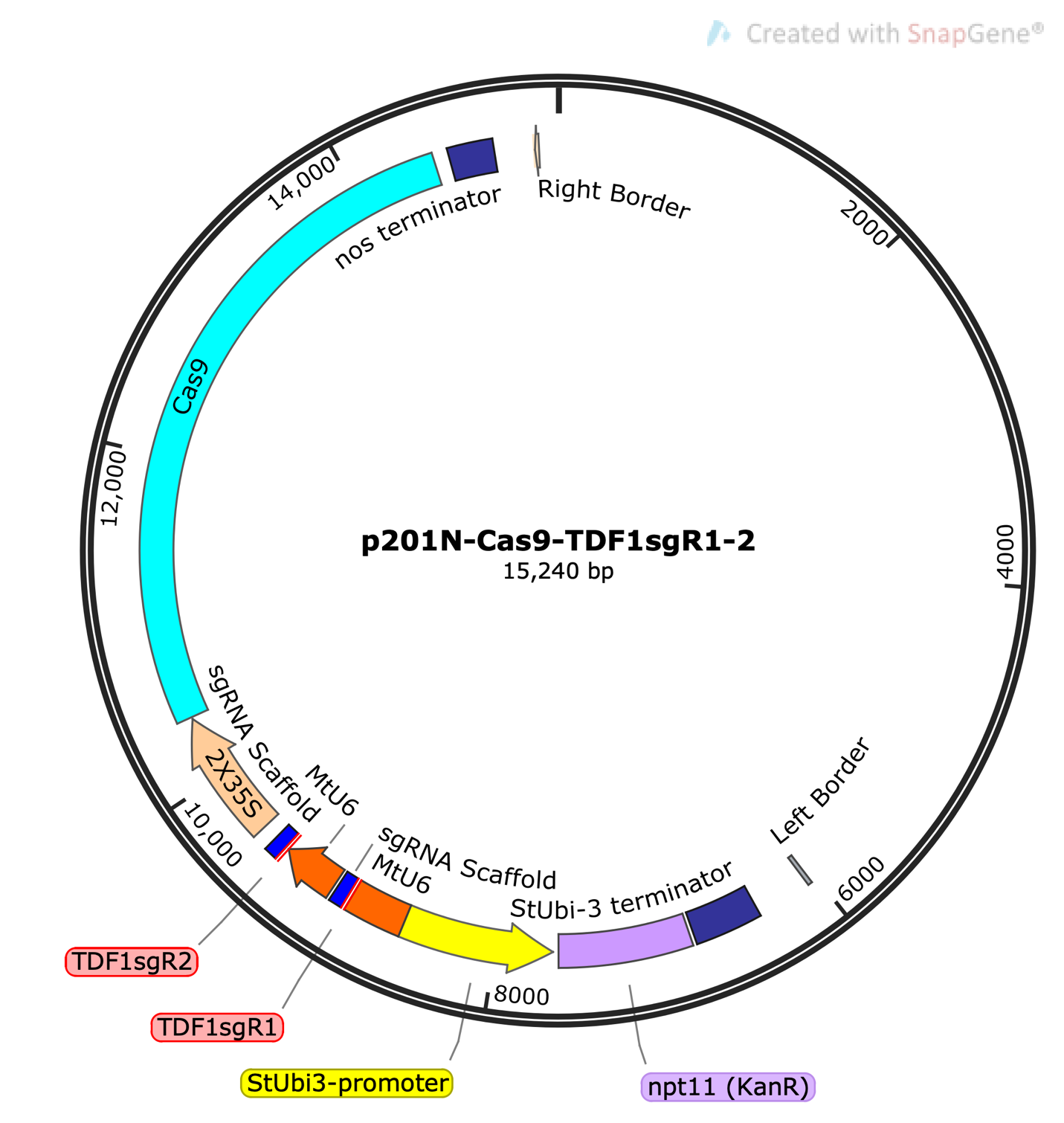


**Supplemental Figure S5. Plasmid map for construct with two *ETDF1* gRNAs and kanamycin-selectable marker.** This figure was prepared in SnapGene and shows key features for the TDNA-integrated region. Cloning was performed as described as described in Methods and Materials, and Jacobs & Martin, 2016. All transformation constructs follow this format, with substitutions for sgRNAs and selectable markers as described.


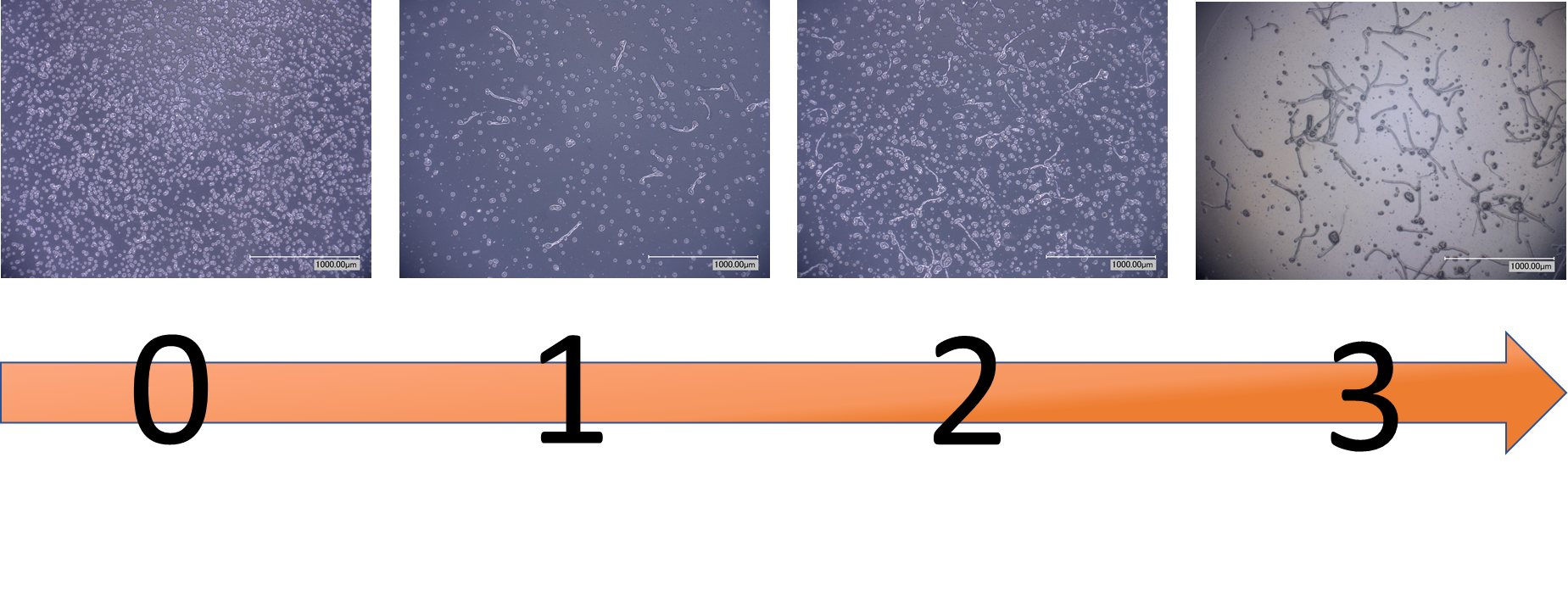


**Supplemental Figure 6. Scoring system for pollen germination**. The extent of germination of pollen in a given sample was scored on a scale of 0 (no visible germination) to 3 (extensive germination), with examples for each score shown in this figure. All samples were observed under 100x magnification.


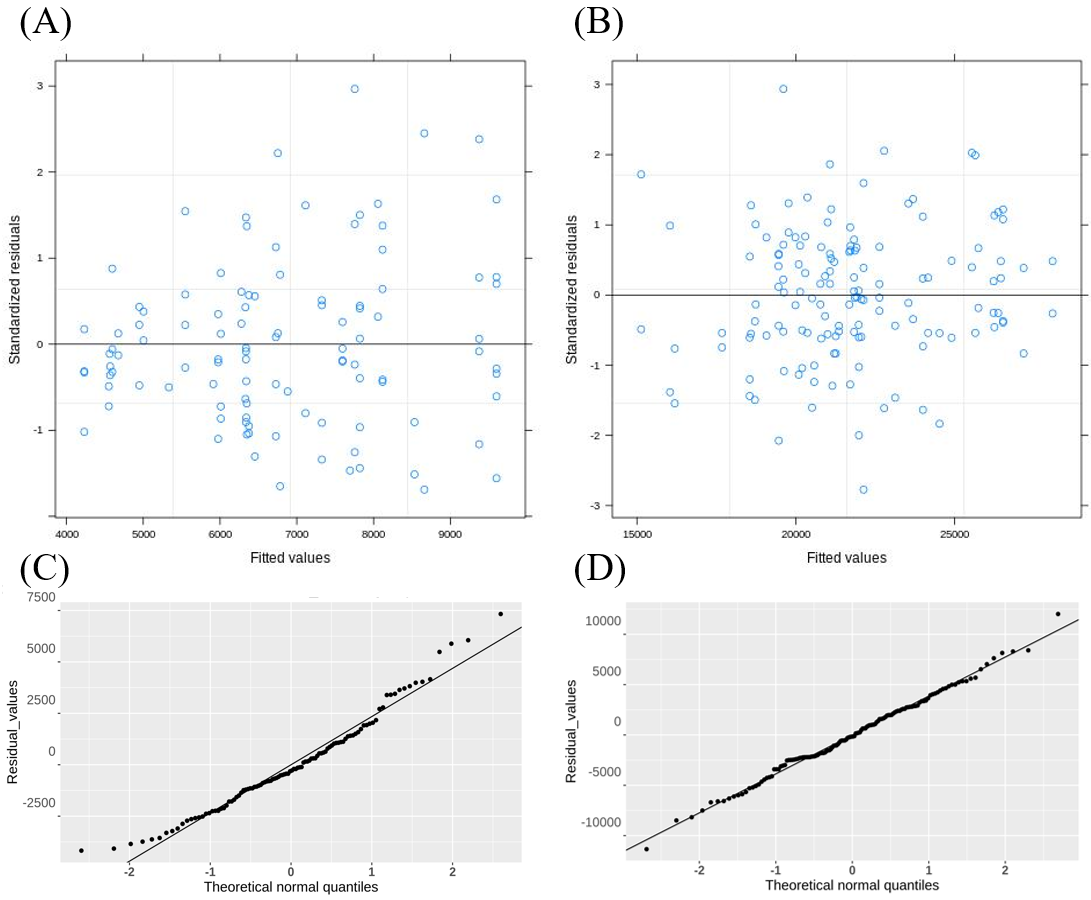


**Supplemental Figure 7. Residual plots for final volume index before log transformation.** Scedasticity was assessed with scattergrams of standardized residuals against fitted values (top) and with Q-Q plots of residual values against a theoretical normal distribution with the same mean and variance (bottom). (A) Standardized residuals vs. fitted values for the group of plants with *etdf1*, *ehec3-like* and controls. (B) Standardized vs. fitted values for the group of plants with e*rec8* and controls. (C) Normal Q-Q plot of the group of plants with *etdf1*, *ehec3-like* and controls. (D) Normal Q-Q plot of the group of plants with e*rec8* and controls.


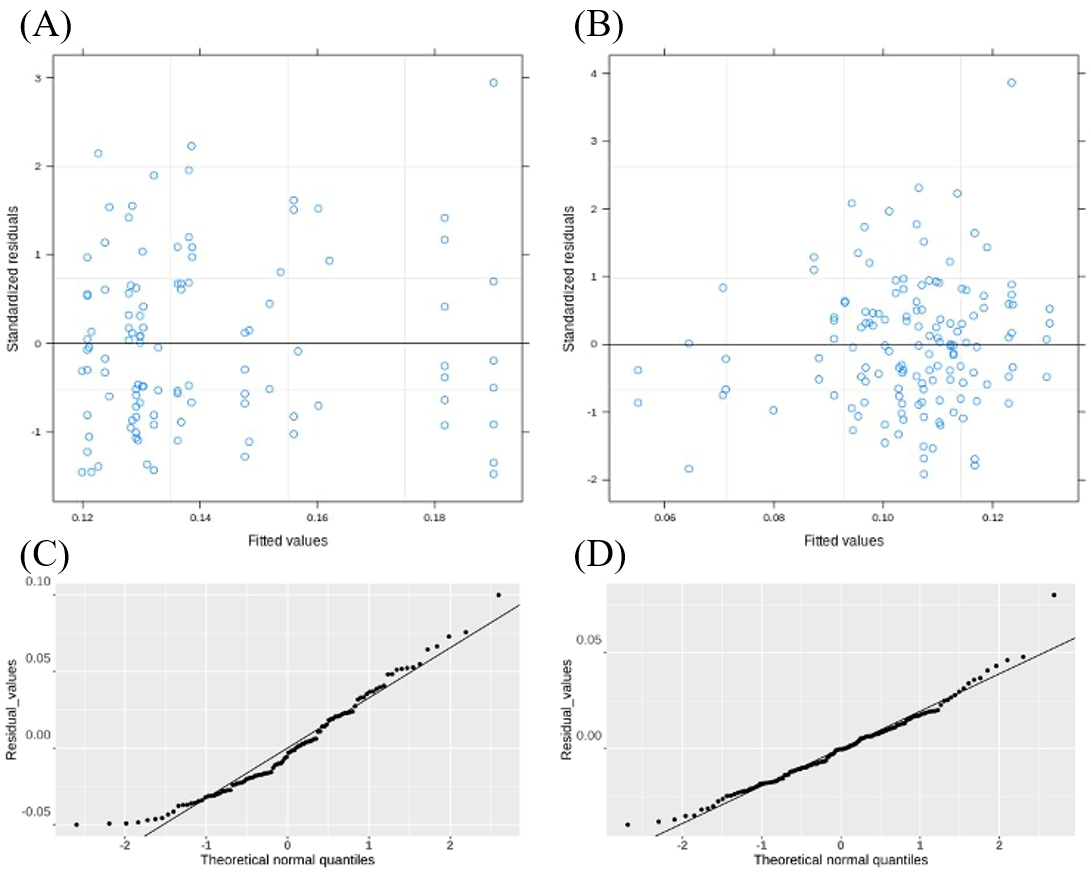


**Supplemental Figure 8. Residual plots for leaf mass before log transformation.** Scedasticity was assessed with scattergrams of standardized residuals against fitted values (top) and with Q-Q plots of residual values against a theoretical normal distribution with the same mean and variance (bottom). (A) Standardized residuals vs. fitted values for the group of plants with *etdf1*, *ehec3-like* and controls. (B) Standardized vs. fitted values for the group of plants with e*rec8* and controls. (C) Normal Q-Q plot of the group of plants with *etdf1*, *ehec3-like* and controls. (D) Normal Q-Q plot of the group of plants with e*rec8* and controls.


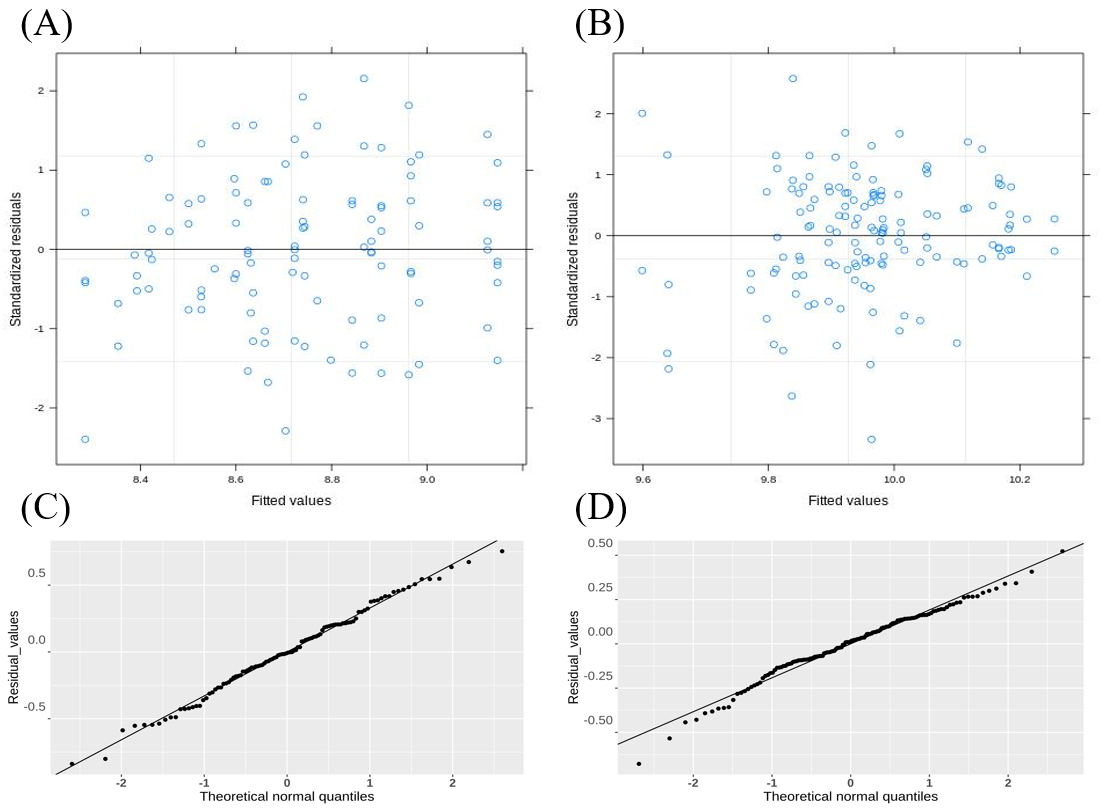


**Supplemental Figure 9. Residual plots for volume index after log transformation.** Scedasticity was assessed with scattergrams of standardized residuals against fitted values (top) and with Q-Q plots of residual values against a theoretical normal distribution with the same mean and variance (bottom). (A) Standardized residuals vs. fitted values for the group of plants with *etdf1*, *ehec3-like* and controls. (B) Standardized vs. fitted values for the group of plants with e*rec8* and controls. (C) Normal Q-Q plot of the group of plants with *etdf1*, *ehec3-like* and controls. (D) Normal Q-Q plot of the group of plants with e*rec8* and controls.


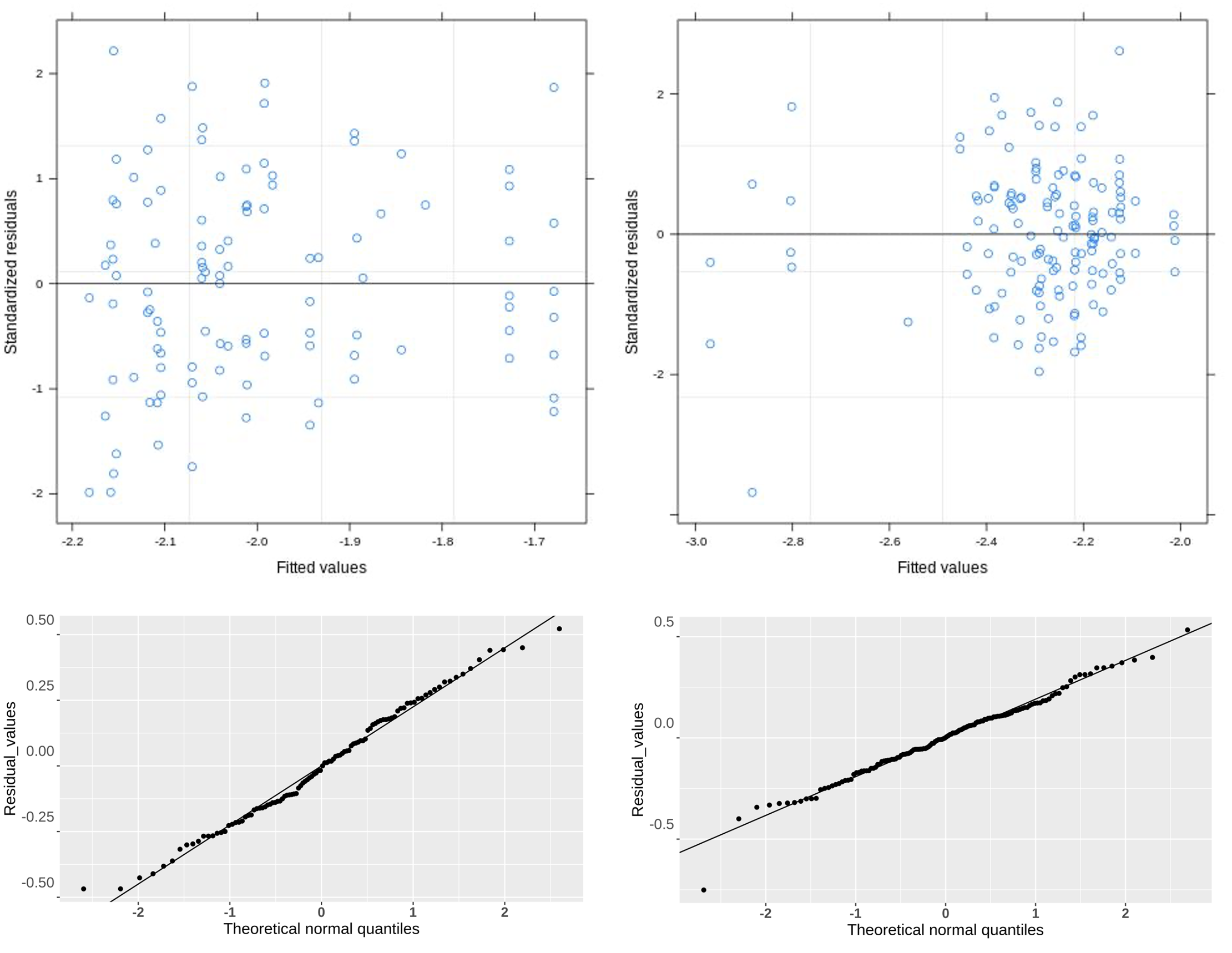


(A) (B)

(C) (D)

**Supplemental Figure 10. Residual plots for leaf mass after log transformation.** Scedasticity was assessed with scattergrams of standardized residuals against fitted values (top) and with Q-Q plots of residual values against a theoretical normal distribution with the same mean and variance. (A) Standardized residuals vs. fitted values for the group of plants with *etdf1*, *ehec3-like* and controls. (B) Standardized vs. fitted values for the group of plants with e*rec8* and controls. (C) Normal Q-Q plot of the group of plants with *etdf1*, *ehec3-like* and controls. (D) Normal Q-Q plot of the group of plants with e*rec8* and controls.


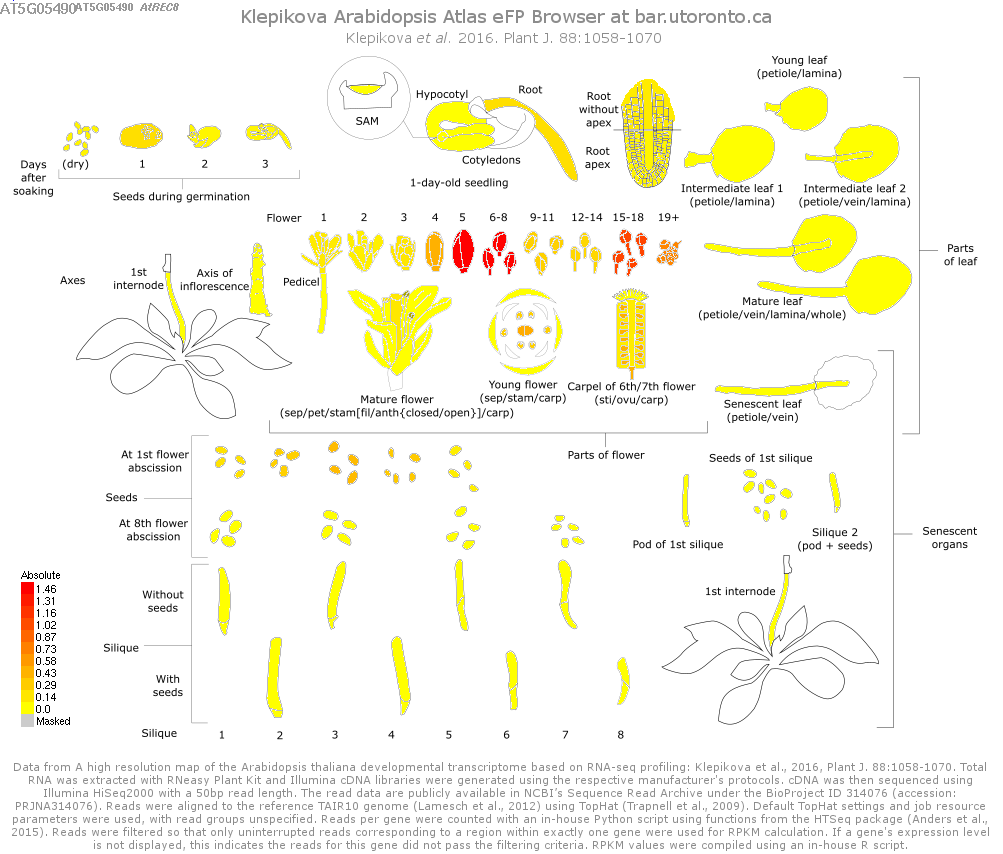


**Supplemental Figure 11. Expression of *AtREC8*, also known as *AtSYN1*.** This figure was produced using the Arabidopsis eFP Browser (bar.utoronto.ca).


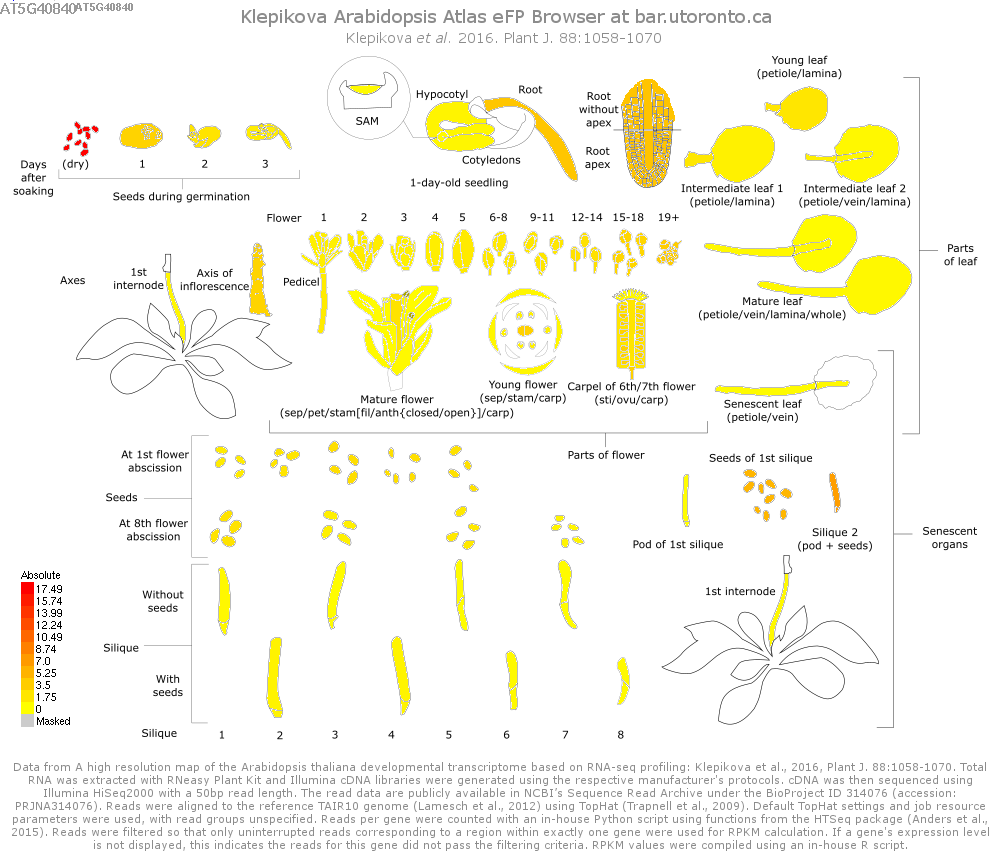


**Supplemental Figure 12. Expression of *AtSYN2***. This figure was produced using the Arabidopsis eFP Browser (bar.utoronto.ca).


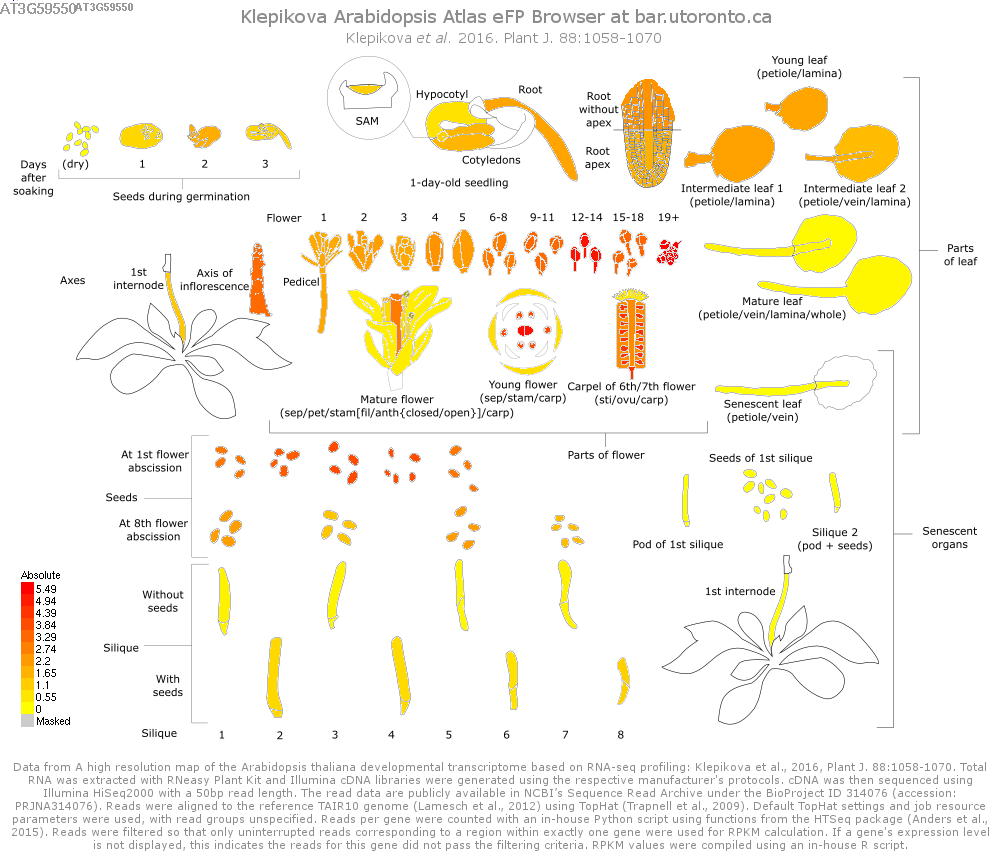


**Supplemental Figure 13. Expression of *AtSYN3*.** This figure was produced using the Arabidopsis eFP Browser (bar.utoronto.ca).


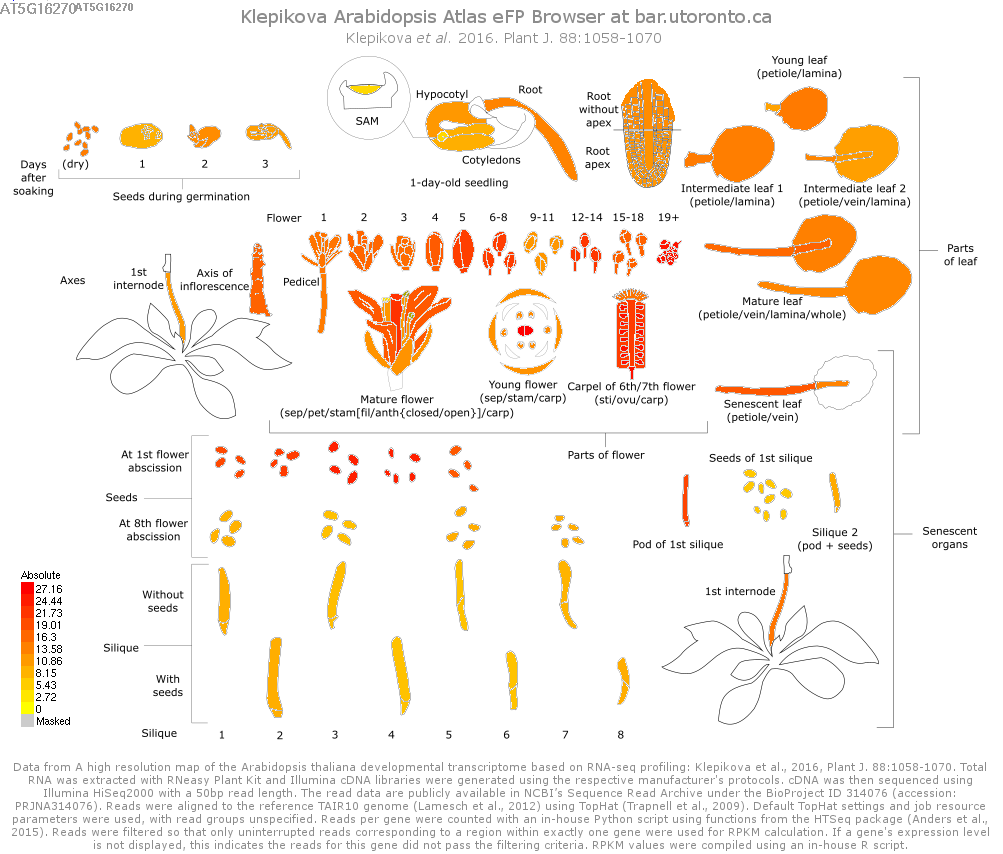


**Supplemental Figure 14. Expression of *AtSYN4***. This figure was produced using the Arabidopsis eFP Browser (bar.utoronto.ca).


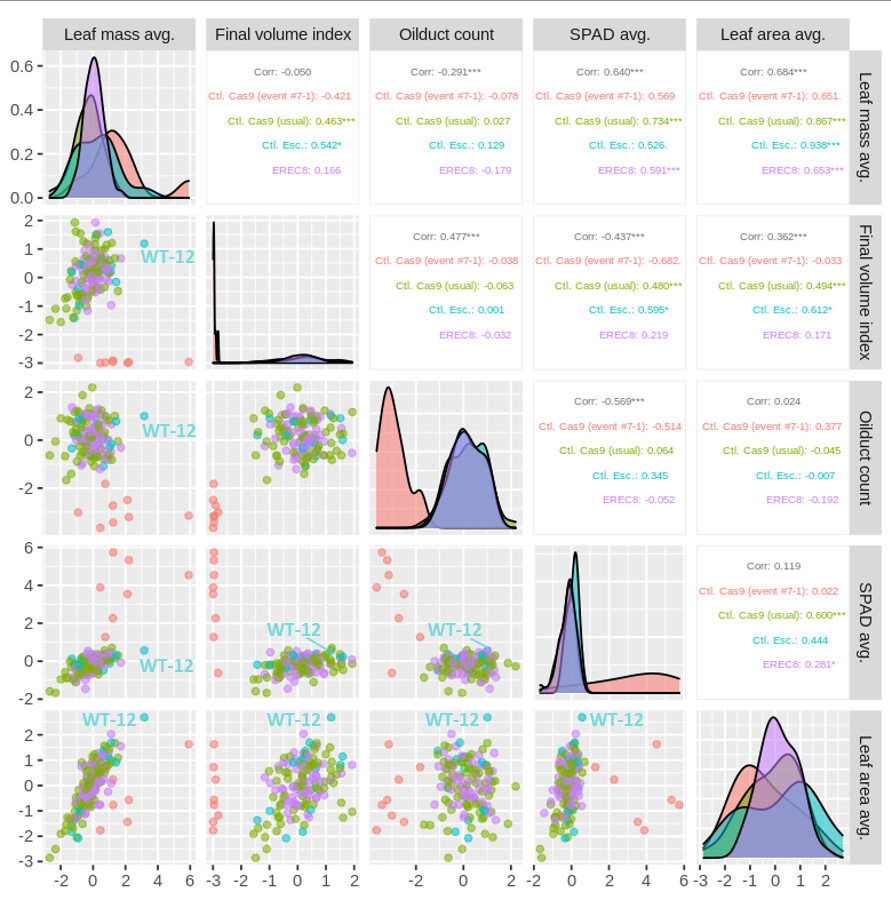


**Supplemental Figure 15. Correlations between traits helped to differentiate outliers**. Plots allow visualization of distinct or extreme values and their relationships across traits. Traits underwent a Z-score transformation prior to plotting to bring them onto a similar scale for easy visualization. This plot was produced with `ggpairs` from the GGAlly package in R. Cas9 control events are divided between event #7-1, which has eight ramets with distinct values (red), and all other Cas9 control events (green), and thus was excluded from final analyses. A single ramet from the “escape” control group displayed distinct values for leaf mass and leaf area (WT-12) and was thus also excluded from statistical analyses.


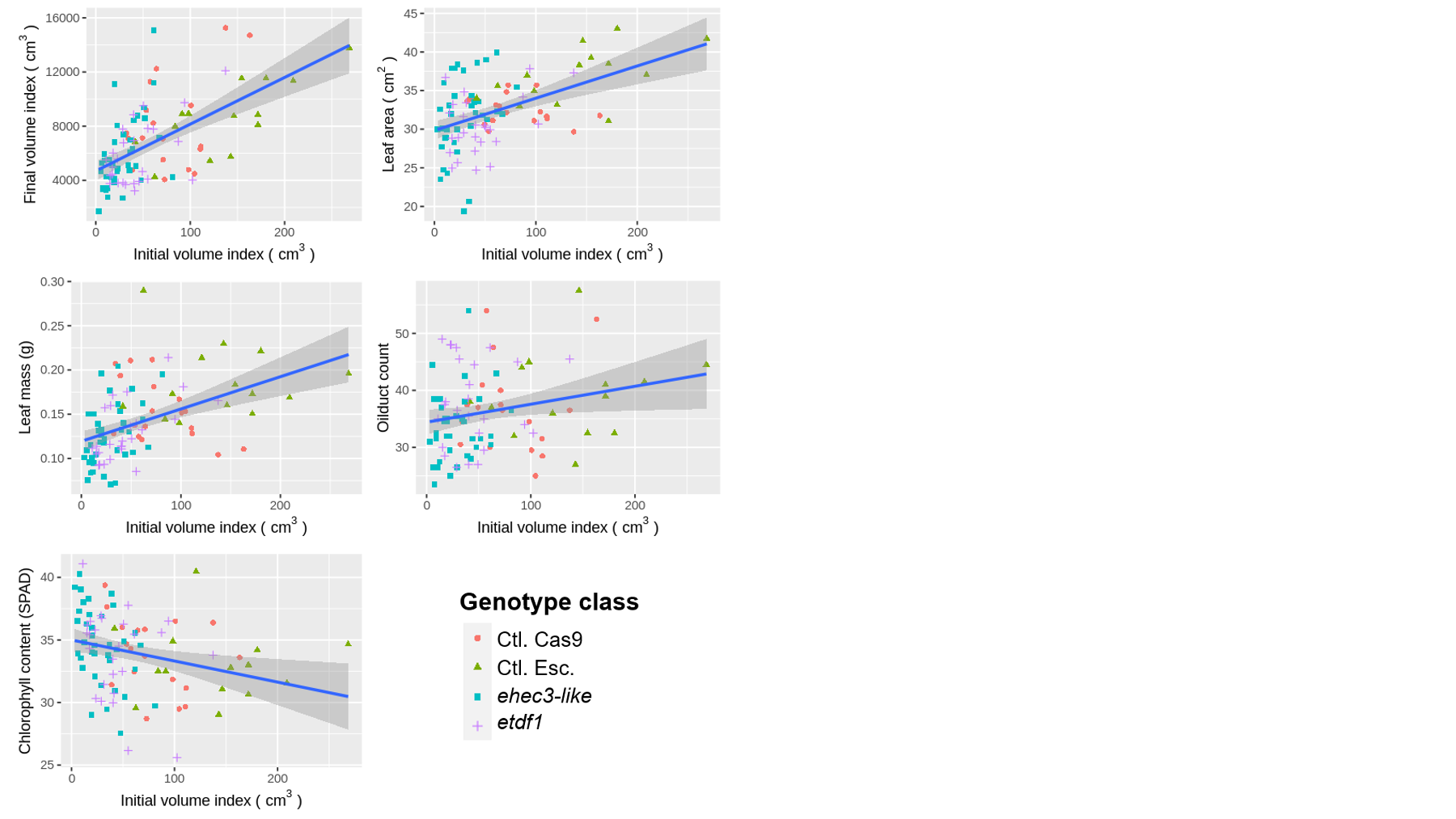


**Supplemental Figure 16. Relationships between initial volume index and traits of interest.** Data shown is from the *etdf1* and *ehec3-like* experimental group, which included control escape (Ctl. Esc.) plants that were transferred to the greenhouse at a different time than experimental lines and were thus of a different size.
