# Supplementary material for: Knockout of floral and meiosis genes using CRISPR/Cas9 produces male‐sterility in Eucalyptus without impacts on vegetative growth
Source: Plant Direct. 2023 Jul 14;7(7):e507. doi: 10.1002/pld3.507 (PMC10345981; doi:10.1002/pld3.507)
Supplement: Supplementary file 2 — Data S2. Supporting Information. [file PLD3-7-e507-s009.docx]

**DECISION LETTER- Round 1**

February 28, 2023

Steven H. Strauss
Oregon State University
Department of Forest Science, Richardson Hall
Corvallis, Oregon 97331-5752


RE: Knockout of floral and meiosis genes using CRISPR/Cas9 produces male-sterility in Eucalyptus without impacts on vegetative growth

Dear Dr. Strauss:

Thank you for submitting to Plant Direct. All required reviews have been returned and we have now finished our evaluation of your manuscript. In light of the reviewers' and editor's comments, further revisions are needed before the paper can be accepted for publication in Plant Direct.

Please view the editors' and reviewers' comments below and use their suggestions as a guide while you work on your revision.

When uploading the revised version of this article, please be sure to include the following:

-A word document that contains your response to the reviewers. You should respond to each reviewer comment and note the changes made to the manuscript. If you do not agree with a reviewer's comment and choose not to make a suggested revision, please explain why. Please try to provide as complete an answer as possible to each reviewer's criticisms in the "response to reviewers" section.
-A tracked changes document
- A clean version of the latest version of the manuscript


To upload your revision, please click the link below.
https://plantdirect.msubmit.net/cgi-bin/main.plex?el=A7Lr6Yt6A7gf6I6A9ftd2QiCC1M73PTpnzfq62ReQZ


In order to provide as timely a service as possible, we ask that your revision is resubmitted within three months after receipt of this request. If an extension is needed, please send a request, along with a brief explanation, to the editorial office at plantdirect@wiley.com .


Thank you very much for giving us an opportunity to review your work. I look forward to receiving the next version.


Sincerely,
Jose Seguí-Simarro


---------------------------------------------------------------------------- Editor comments:


---------------------------------------------------------------------------- Reviewer comments:
Reviewer #1:

The reviewer suggests different suggestions in the attached file. Hope for an improved version.


Reviewer #2:

In this manuscript, the author knockout EREC8, ETDF1 and EHEC3-Like by CRISPR/Cas9 approach in Eucalyptus spp. The author described the identification of homologous genes in Eucalyptus spp by sequence alignment, investigated the vegetative growth of transgenic plants, and simply observed the pollen formation and germination phenotype of transgenic plants. Overall, I think the structure of this manuscript is chaotic and lacks enough evidences to support their viewpoints.
1) Some description of results is irrelevant with the title of this manuscript. For example, the author described a lot about how to obtain homologous proteins and the effectiveness of their CRISPR/Cas9 approach in the results. However, they did not perform detailed analysis for the obtained CRISPR lines. In addition, the title of this manuscript is 'Knockout of floral and meiosis genes using CRISPR/Cas9 produces male-sterility in Eucalyptus...', but CRISPR mutants of two targets genes may have both male and female sterile phenotype. 2) The structure of this manuscript is chaotic. Figure 1 and Figure 2 showed the sgRNA sites and schematic of experimental design in the methods section. If the author thinks this is a very important result, please put it in the results section. 3) The information about mutation sites at these CRISPR lines are not shown to the readers. 4) For the determination of homologous proteins, the author only used the sequence alignment, but not analyzed their expression pattern in Eucalyptus spp. with such as RT-PCR or in situ hybridization. 5) For the obtained CRISPR lines, only observation of visible pollen and staining was not enough. For the etdf1 mutants, further cytological analysis of anther section is necessary. For the erec8 lines, further DAPI staining is also feasible to check whether the meiosis is abnormal. In addition, the author said that 'erec8 Eucalyptus will be female- as well as male-sterile'. Why did the author not perform hybridization to verify this phenotype? For the phenotype of ehec3, it is distinctly different from the previously reported phenotype of Arabidopsis homologue mutants. What is the mechanism of ehec3 function for Eucalyptus pollen development? Based on these concerns, I therefore think that the manuscript is not suitable for publication in Plant Direct.


Reviewer #3:

This work documents the generation and phenotypic characterization of eucalyptus trees in which CRISPR/Cas9 technology disrupted reproductive genes.
Some recommended minor revisions:
1 - Lines 518-522 - Is it possible that the high rate of pollen abortion seen in the erec8 control events was due to the lengthy storage of anthers prior to viability staining (up to one month, lines 288-289)? It is worth mention that the high rate of control pollen abortion (Table S13) and the low rates of control pollen germination, 0 in some cases (Table S15) do make it difficult to assess the full potential of erec8 mutations to compromise pollen viability.
2 - Can the authors clarify the liquid media that was used to recover the pollen for germination assays (lines 317-323)?

**DECISION LETTER- Round 2**

June 1, 2023
Prof. Steven Steven Henry Strauss Strauss
Oregon State University
Department of Forest Ecosystems and Society, Richardson Hall
3322 SW Cascade Ave
Corvallis, Oregon 97333


MSID: 2022-01101R1
MS TITLE: Knockout of floral and meiosis genes using CRISPR/Cas9 produces male-sterility in Eucalyptus without impacts on vegetative growth

Dear Dr. Steven Strauss:

I am pleased to inform you that your manuscript "Knockout of floral and meiosis genes using CRISPR/Cas9 produces male-sterility in Eucalyptus without impacts on vegetative growth" has been accepted for publication in Plant Direct.

Your article will appear online in the next available issue of Plant Direct. To ensure your article gets published as quickly as possible, please pay attention to the steps detailed below. We have found that most of the delays happen at this stage, especially at the payment stage, so please respond as quickly as possible when prompted.

License Agreement: Once your article has been accepted it will move to Production and undergo admin and file checking - you may receive an email with any queries we have at this stage. When all required items are received by the publisher and queries resolved, the corresponding author will receive an email from Wiley's Author Services system which will ask them to log on at https://authorservices.wiley.com/bauthor and will present them with the appropriate license for completion. Your article cannot be published until both the signed license agreement and payment of the article fee have been received.

Payment of the Open Access Article Publication Fee: All articles published in Plant Direct are fully open access: immediately and freely available to read, download and share. Plant Direct charges a publication fee to cover the publication costs. The corresponding author for this manuscript should have already received a quote from the payments team (cs-openaccess@wiley.com) with the estimated article publication fee; please email cs-openaccess@wiley.com if this has not been received. The corresponding author should log on to the Wiley Author Services site, where the publication fee can be paid by credit card or an invoice. Pro Forma can also be requested. Payment of the publication charge must be received before the article will be published online.

Proofs: You will have the opportunity to look over your paper once more when you receive the author proofs for your article. The proofs will be with you in approximately two weeks. Please note that, in addition to publishing reviewer comments, the author's responses to review comments will also be published alongside the final version of the paper. If you would not like the author's responses to be published, please contact the editorial office at plantdirect@wiley.com .

Promotion of your article: You can help your research get the attention it deserves! Check out Wiley's free Promotion Guide for best-practice recommendations for promoting your work at www.wileyauthors.com/eeo/guide. And learn more about Wiley Editing Services which offers professional video, design, and writing services to create shareable video abstracts, infographics, conference posters, lay summaries, and research news stories for your research at www.wileyauthors.com/eeo/promotion.

Thank you again for your contribution to Plant Direct. If you have any questions, feel free to contact the editorial office at plantdirect@wiley.com .

Sincerely,

Jose Seguí-Simarro

Hsou-min Li

Editor, Plant Direct


---------------------------------------------------------------------------- Editor comments
The authors have have addressed fairly well almost all the issues raised by the reviewers. For issues not possible to solve, explanations have been given and the text has been modified to highlight the importance of future studies in this direction.


---------------------------------------------------------------------------- Reviewer comments:

--

AUTHORS’ RESPONSE

Dear Dr. York and Dr. Seguí-Simarro:

We wish to express our gratitude to both of you, and to all the reviewers, for the many detailed and helpful comments; as you can see below and in the uploaded manuscript with revision marks and comments, we have responded to all of them.

Perhaps the major issue raised was the desired for further research on the mechanisms of the candidate genes by reviewer 2. As you may recall, via emails we argued that this is beyond scope and impossible because the trees are no longer available, and you agreed. We are pleased that you both agreed (the emails are quoted below for your convenience). However, to guide further research we did propose specific follow-on studies for each of the candidate genes.

Below, we provide explanations of the revisions made in response to feedback from each reviewer; more details can be found in the comments made in the resubmitted manuscript.
There, specific comments are provided in-line with text, along with our responses.

***Reviewer 1***

Reviewer 1 had a number of helpful comments on writing and format, and also made several requests for additional detail. We appreciate these suggestions, and have adopted many of them. We explain our general responses to the major issues below.

- Various comments suggested that we avoid the active voice and self-reference (e.g., “we performed…”) and to instead use the passive voice (e.g., “In the current study, flowers were…”) throughout the manuscript. Currently, the manuscript uses a mix of the active and passive voice, and includes self-reference (“we”) for the sake of conciseness and readability. We chose to, in most cases, retain the current writing style as we felt it was most concise and readable.
- The reviewer made many additional suggestions to revise writing, several of which we’ve incorporated (e.g., using the term “candidate gene” instead of “gene candidate”). However, we did not incorporate others in order to maintain a consistent and clear narrative voice.
- Various comments suggested that we avoid all use of the present-tense. We have aimed to be conservative in using present tense only when referring to the already-published and established roles of genes that continue to exist in the present. To this end, we have made several changes in the third paragraph of the introduction to use the past tense where we refer to our Eucalyptus gene candidates which have not already been studied in published literature. However, in the fourth paragraph when referring to established functions of Arabidopsis homologs and the established nature of these genes, which continue to exist, we have chosen to use the present tense.
- The reviewer suggested adding references for various pieces of equipment and software methods used throughout the study. We have revised to ensure that the companies who produced each piece of equipment are clearly stated. We have added a peer-reviewed reference for ImageJ. We made clear which analyses were done with various statistical environments, including base R and the R package `emmeans,` and although we do not have a peer-reviewed reference for this package it can be found by its name. We have also added a reference for the plant acclimation protocol involving placing Ziploc bags over plants.
- The reviewer suggested at several point to use lowercase “l” for units including mL and µL. We prefer to use the uppercase “L” for readability purposes and in accordance with guidelines from the U.S. National Institute of Standards and Technology guidance (<https://www.nist.gov/pml/owm/writing-si-metric-system-units>)

***Reviewer 2***

As cited above, reviewer 2 highlighted a number of areas to provide further details and suggested that additional experiments are desired to provide mechanistic insights into our candidate genes before the manuscript would be suitable for publication. We appealed this decision, to which Dr. York wrote, “Typically, I don’t see our role as to demand new experiments (I don’t request this when reviewing or editing for ANY journal personally), though I do think new data analysis requests are reasonable. I see our role as to make sure the results presented have necessary context, including limitations and caveats of the data, and what is speculation,” and Dr. Seguí-Simarro wrote, “if the trees are gone, it is evident that pollen characterization is not possible. I will not request it.” Instead, we made extensive additions to the Results/Discussion to better provide context on the limitations of our findings, and to outline future directions for research, including cytological work to characterize candidate genes’ mechanisms, field studies and seed germination assays. We also expanded the discussion to describe suggested next steps for mechanistic characterization of the gene candidates, and modified the Methods and Results/Discussion to make clear that detailed analysis of CRISPR/Cas9-mediated mutations was performed and is given in Table S3.

***Reviewer 3***

Reviewer 3 made requests for additional technical details and asked for clarifications on the limitations of our pollen germination assay. We have revised the manuscript to fully address these concerns.

This reviewer was also concerned about our level of detail and interpretation of results for *erec8* phenotypes, as well as the availability of methods for the pollen germination assay. We have made extensive revisions to the *Results/Discussion* on *erec8* phenotype to address feedback from all reviewers, and have also clarified methods for the pollen germination assay (both in text and Table S13).

***Editor comments***

Dr. Seguí-Simarro noted, “all along the manuscript, including the figure legends, different typos and mistakes have been found and should be corrected.” Although the material we received did not highlight any of these in particular aside from those noted by Reviewer 1, we have performed an additional round of proofreading and believe we have found and corrected all of these where appropriate.
